# Progress on the Attenuation Mechanism and Modification of the Cobalt‐Free Spinel LiNi_0.5_Mn_1.5_O_4_

**DOI:** 10.1002/advs.202508121

**Published:** 2025-07-21

**Authors:** Bao Zhang, Zhen Liang, Peng Dong, Weili Song, Yiyong Zhang, Xue Li, Xiaoyuan Zeng, Yongkang Liu, Ziliang Feng, Enfeng Zhang, Yingjie Zhang, Yannan Zhang

**Affiliations:** ^1^ National and Local Joint Engineering Research Center for Lithium‐ion Batteries and Materials Preparation Technology Key Laboratory of Advanced Battery Materials of Yunnan Province Faculty of Metallurgical and Energy Engineering Kunming University of Science and Technology Kunming 650093 China; ^2^ Institute of Advanced Structure Technology Beijing Institute of Technology Beijing 100081 China

**Keywords:** attenuation mechanism, cobalt‐free high‐voltage cathode material, lithium‐ion battery, modification strategy, spinel LiNi_0_._5_Mn_1_._5_O_4_ (LNMO)

## Abstract

Cobalt‐free LiNi_0.5_Mn_1.5_O_4_ (LNMO) has recently emerged as a highly promising cathode material owing to its benefits of a high operating voltage platform (≈4.7 V vs Li), high theoretical energy density (≈650 Wh kg^−1^), eco‐friendliness, and resource abundance. However, it has also demonstrated low cycle and poor rate performances. Researchers have hitherto identified multiple LNMO failure and degradation mechanisms, including the Jahn‐Teller effect, Transition Metal (TM) dissolution, electrolyte decomposition, and Oxygen Vacancies (OVs). The Jahn‐Teller effect causes structural material degradation, while TM dissolution could lead to the loss of reactive species and interfacial side reactions. On the other hand, OVs and electrolyte decomposition accelerate capacity decay. Notably, deeply understanding LNMO structural failure mechanisms and the targeting of corresponding modifications presents a vital avenue for modulating its surface‐interface structure and improving its electrochemical performance. Although researchers have extensively investigated the failure mechanisms of LNMO to elucidate its modification strategies, a comprehensive and detailed summary of the latest research advancements has yet to be provided. In this work, the research background, encompassing the advantages and disadvantages of LNMO cathode materials, is first introduced. The crystal structure and discharge mechanisms, among other fundamental principles of LNMO, are subsequently analyzed. Finally, recent research findings on the aforementioned failure mechanisms in high‐voltage spinel LNMO are synthesized. Subsequently, a critical assessment of recent advancements in modification strategies targeting the failure mechanisms of LNMO is performed, encompassing the tools employed (e.g., doping modification, surface coating, morphology and size management, and surface orientation management) as well as their synergistic effects. Finally, potential future research directions to guide the rational design of high‐performance LNMO, particularly manganese‐based spinel cathode material, are proposed.

## Graphical abstract

This review provides an overview of the development history, working mechanisms, and scientific challenges of LiNi_0.5_Mn_1.5_O_4_ cathode materials for Lithium‐ion battery and discusses the strategies to improve the electrochemical performance of LiNi_0.5_Mn_1.5_O_4_.

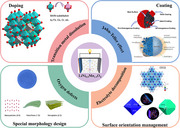

## 1 Introduction

**Figure 1:**
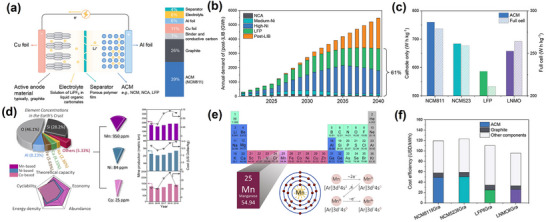
a) A schematic illustration of a LIBs cell and the weight fractions of the cell components. Reproduced with permission.^[^ ^13b^ ^]^ Copyright 2024, Wiley‐VCH GmbH. b) The predicted demand of LIBs and post‐LIBs. Reproduced with permission.^[^ ^14^ ^]^ Copyright 2023, Nature Energy. c) Breakdown of different LIB pack cost per kWh. Reproduced with permission.^[^ ^13b^ ^]^ Copyright 2024, Wiley‐VCH GmbH. d) Advantages of Mn compared with Ni and Co, in terms of crust abundance, characteristics of LiTMO_2_ (TM = Mn, Ni, Co), and annual mine productions and costs. Reproduced with permission.^[^ ^12^ ^]^ Copyright 2021, Elsevier Inc. e) The atomic structure of Mn and its typical valence states in cathode materials. Reproduced with permission.^[^ ^12^ ^]^ Copyright 2021, Elsevier Inc. f) Material‐level and cell‐level energy densities of LNMO compared to mainstream ACMs. Reproduced with permission.^[^ ^13b^ ^]^ Copyright 2024, Wiley‐VCH GmbH.

Recently, there has been considerable growth in the global demand for renewable energy technologies to help address issues such as energy depletion and environmental pollution.^[^ ^1^ ^]^ With the global transition to clean energy gaining momentum in several countries, there has been an urgent need to develop eco‐friendly, safe, and reliable power battery systems with higher energy density.^[^ ^2^ ^]^ There is an urgent global demand for renewable clean energy technologies, as well as compatible energy conversion and storage systems.^[^ ^3^ ^]^ Owing to attributes such as cleanliness and high efficiency, chemical power systems are increasingly becoming indispensable in modern society.^[^ ^4^ ^]^ Lithium‐Ion Batteries (LIBs) are the most popular energy storage devices in portable electronic products, electric vehicles, and smart grid systems—a phenomenon attributable to their high energy density and long cycle life.^[^ ^5^ ^]^ LIBs mainly comprise a cathode, an anode, a diaphragm, and an electrolyte (**Figure** **1**a).^[^ ^6^ ^]^ Among these factors, the cost and performance of cathode materials play a crucial role in determining the overall performance of LIBs, with the charge/discharge platform and specific capacity serving as the primary determinants of the battery's energy density.^[^ ^7^ ^]^ Recently, the development of cathode materials has recently gained increasing scholarly attention.^[^ ^8^ ^]^

Traditional cathode materials such as LiFePO_4_, LiMn_2_O_4_, LiCoO_2_, and LiMO_2_ (M = Ni, Co, and so on) are commonly used in multiple novel energy platforms (Figure 1b,c).^[^ ^9^ ^]^ Olivine LiFePO_4_ is widely used as a commercial cathode material. However, it cannot meet the demand for high‐performance and low‐cost LIBs due to its low theoretical and actual specific capacity.^[^ ^10^ ^]^ Despite their relatively high theoretical capacity, layered cathode materials demonstrate unsatisfactory utilization of reversible intercalation/deintercalation; these materials are prone to gas evolution reactions and structural degradation during charge/discharge cycles.^[^ ^9d^ ^]^ The resulting safety hazards have also affected their large‐scale commercial promotion.^[^ ^11^ ^]^ Manganese possesses several significant advantages compared to other elements (Figure 1d,e).^[^ ^12^ ^]^ First, it has a significantly higher crustal abundance (950 ppm) than nickel (84 ppm) and cobalt (25 ppm), implying abundant reserves relative to the two. Second, owing to its special atomic/electronic structure, manganese can be used to generate derivatives such as Li_2_MnO_3_, endowing it with a higher capacity and energy density than conventional cathode materials. Manganese also exhibits a lower atomic weight (54.94) than nickel (58.69) and cobalt (58.93), which underscores its potential for higher capacity. Finally, since manganese is also less toxic than nickel and cobalt, it might cause less heavy metal pollution.^[^ ^12^, ^13^ ^]^ Overall, manganese‐based cobalt‐free cathode materials could provide an important resource guarantee and strong support for improving next‐generation high‐performance LIBs. Such developments could also lay the groundwork for the large‐scale development of cobalt‐free cathode materials in the future.

Future research into LIBs will likely focus on energy density, lifetime, cost, and safety.^[^ ^15^ ^]^ Among the numerous cathode materials applicable in LIBs, the cobalt‐free spinel LiNi_0.5_Mn_1.5_O_4_ (LNMO)—due to its low cost and high energy density—could be among the potential candidates for next‐generation, high‐energy‐density LIBs (Figure 1f).^[^ ^16^ ^]^ However, LNMO presents many challenges in the transition from laboratory to practical applications. For instance, the dissolution of transition metals (TMs) may result in the loss of active materials and an increase in interfacial impedance.^[^ ^17^ ^]^ The Jahn‐Teller effect can cause particle rupture and microcracking, thereby diminishing the structural stability of the material.^[^ ^18^ ^]^ Additionally, complex side reactions between the material surface and the electrolyte could generate by‐products that would destroy the material surface structure, hindering ion transport and ultimately causing interfacial destabilization.^[^ ^19^ ^]^ These phenomena could further degrade the performance and stability of batteries.^[^ ^20^ ^]^ Nonetheless, the multiple shortcomings and defects limiting LNMO's further application are likely to be addressed through rapid developments in materials science and related technologies, highlighting their great promise in the realm of Lithium batteries.^[^ ^21^ ^]^ Multiple effective strategies, such as doping modification, surface coating, morphology and size management, and surface orientation management, have recently been proposed to mitigate the Jahn‐Teller effect and suppress transition metal migration and dissolution, thereby enhancing the electrochemical performance of LNMO.^[^ ^22^ ^]^

Compared to nickel and cobalt, manganese is more affordable and less toxic. Therefore, exploring potential cobalt‐free cathode materials based primarily on manganese resources is highly imperative. This article presents a comprehensive overview of representative LNMO cathodes for LIBs. Specifically, besides assessing its advantages and challenges, we describe LNMO development history, fundamentals, and latest research progress. We also examined the latest developments regarding its modification strategies and its current research status. From a research perspective, we review in detail LNMO capacity decay mechanisms, performance improvement methods, and research progress. Finally, we predict the development trend and application prospects of LNMO cathode materials.

### 1.1 Development History and Structure of LNMO

Research on spinel‐structured LNMO dates back half a century. Blasse first described LNMO and a series of its mixed‐metal spinel oxides in 1964, focusing on its crystallography.^[^ ^23^ ^]^ He further explored the magnetic properties of LNMO and other spinel materials after some time.^[^ ^24^ ^]^ Notably, Blaise asserted that the ferromagnetic behavior of LNMO could be somewhat attributed to the crystallographic ordering of paramagnetic Ni^2+^ and Mn^4+^ on octahedral sites. Tarascon et al. first investigated the electrochemical properties of LiNi_y_Mn_2‐y_O_4_ with elemental substitutions in the spinel structure (LiM_x_Mn_2‐x_O_4_, M = Ti, Ge, Fe, and Zn).^[^ ^25^ ^]^ Subsequently, J.R. Dahn et al. charged LiNi_x_Mn_2‐x_O_4_ to 5.0 V for a more comprehensive evaluation of its electrochemical performance.^[^ ^26^ ^]^ With an increasing amount of nickel substitution, the electrochemical plateau observed at 4.1 V gradually weakened, and a new one appeared at 4.7 V, making the capacity of LiNi_x_Mn_2‐x_O_4_ consistent with that of LMO. Moreover, the electrochemical plateau at 4.7 V correlated with the Ni^2^⁺/⁴⁺ redox, and its electron binding energy (0.6 eV) was higher than that of Mn electrons. It is also noteworthy that the electrochemical plateau at 4.7 V dominated the electrochemical behavior of LiNi_0_._5_Mn_1_._5_O_4_ at 0.5 Ni content.

### 1.2 Structure of LNMO

**Figure 2:**
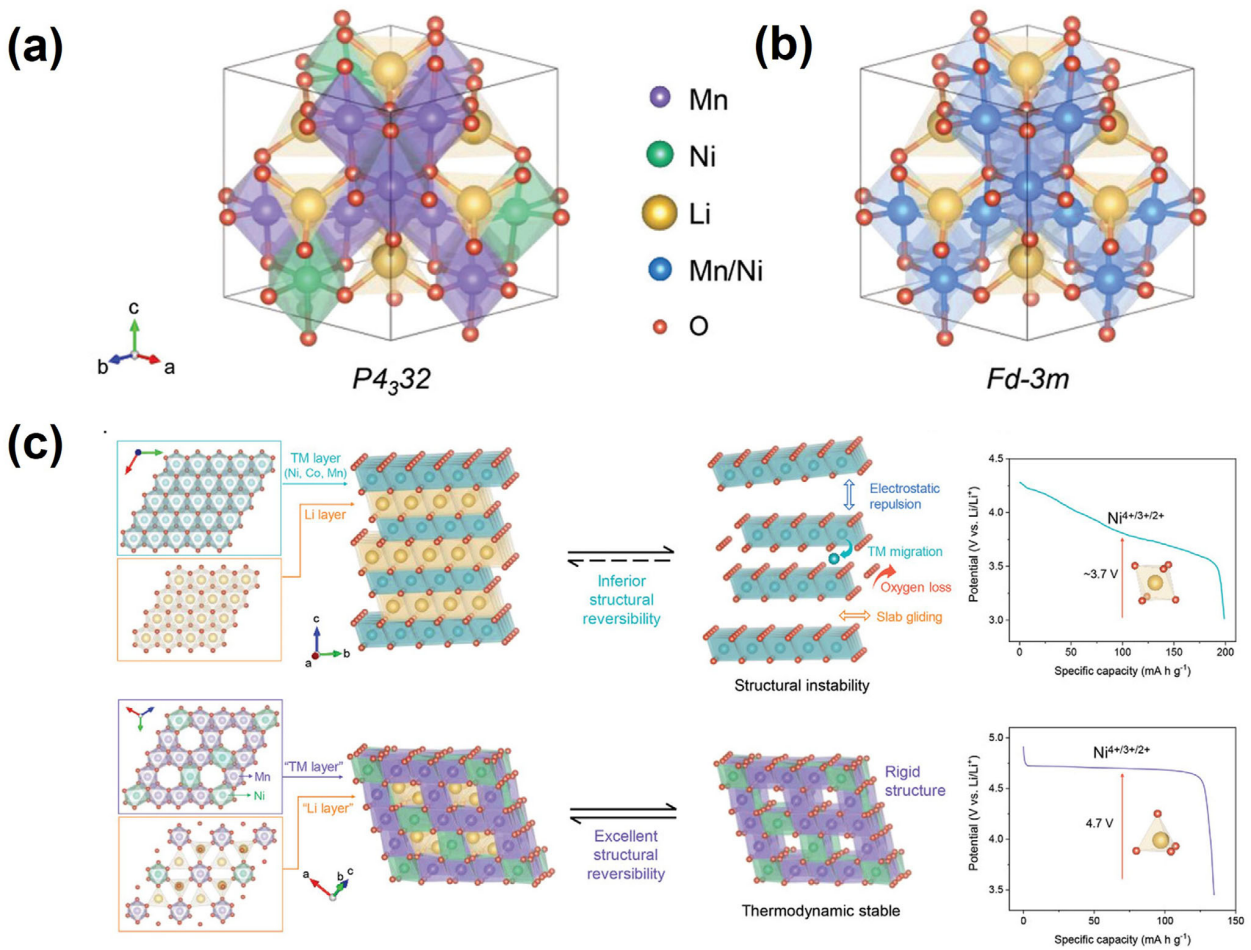
Crystal structures of a) LNMO (*P4_3_32*) and b) LNMO (*Fd3m*). Reproduced with permission.^[^ ^13b^ ^]^ Copyright 2024, Wiley‐VCH GmbH. c) Comparison of spinel LNMO with typical nickel‐based NCMs regarding the structure and electrochemical properties. Reproduced with permission.^[^ ^13b^ ^]^ Copyright 2024, Wiley‐VCH GmbH.

There are two possible space group structures for LiNi_0.5_Mn_1.5_O_4_. First, Ni replaces part of Mn, occupying the 16d site, thus forming M (Ni, Mn)─O bonds. The resultant belongs to the *Fd3m* space group and assumes the face‐centered cubic structure of LiNi_0.5_Mn_1.5_O_4‐δ_, where δ is the oxygen defect content (**Figure** **2**b). Second, Ni replaces part of Mn, occupying the 4a position while Mn occupies the 12d position, forming Ni─O and Mn─O bonds, respectively. The resultant belongs to the *P4_3_32* space group and assumes the ordered cubic structure of LiNi_0.5_Mn_1.5_O_4_ (Figure 2a).^[^ ^27^ ^]^

In the disordered *Fd3m* structure, Li migrates from the tetrahedral 8a site to the 16c vacancy, creating the 8a‐16c diffusion path. In the ordered *P4_3_32* structure, octahedral 16c vacancies are distributed across the octahedral 4a and 12d sites in a 1:3 ratio, yielding the 8c‐4a and 8c‐12d diffusion paths, respectively.^[^ ^13^, ^28^ ^]^ The coulomb potentials of the three diffusion paths are reported to follow the order of 8c‐4a < 8a‐16c < 8c‐12d.^[^ ^29^ ^]^ Both the 8c‐4a (the easiest diffusion path) and 8c‐12d (the hardest diffusion path) are detected in the ordered spinel structure.^[^ ^30^ ^]^ However, the most favorable diffusion path in the ordered *P4_3_32* structure only occupies 25% of the total diffusion path, making the disordered *Fd3m* structure more favorable for Li^+^ diffusion.^[^ ^31^ ^]^ Regarding electronic conductivity, it is noteworthy that Mn^3^⁺ presence in a disordered spinel contributes to its conductivity. The conductivity of the disordered structure was found to be 10⁻⁴^.^⁵ S cm⁻¹, 2.5 orders of magnitude higher than that of the ordered structure.^[^ ^32^ ^]^ In addition, the generation of NiMnO_3_ and α‐Mn_2_O_3_ from the disordered structure is accompanied by a large amount of oxygen generation during the charging and discharging process. In the ordered structure, the Ni/Mn ordering lowers the ionic mobility barrier, resulting in the formation of the high‐temperature resistant NiMn_2_O_4_ phase and the release of a small amount of oxygen. The structural stability of the Mn^4+^ octahedron is superior to that of the Mn^3+^ octahedron. Additionally, the transformation from ferromagnetic Mn^4+^‐ O^2 ‐^‐ Mn^4+^ into the antimagnetic Ni^2+^‐ O^2 ‐^‐ Mn^4+^ further enhances the structural stability of the material. Therefore, the thermal stability of the ordered structure is superior to that of the disordered one.^[^ ^33^ ^]^

### 1.3 The Electrochemical Behavior of High‐Voltage LNMO

Different space groups in LNMO exhibit distinct behaviors in charging and discharging processes.^[^ ^34^ ^]^ The disordered *Fd3m* structure shows a small plateau at ≈4.0 V, corresponding to the Mn^4+^/Mn^3+^ redox reaction of. Two additional plateaus appear at ≈4.6 V and ≈4.8 V, corresponding to the Ni^3+^/Ni^2+^ and Ni^4+^/Ni^3+^ redox reactions, respectively.^[^ ^33b^ ^]^ In contrast, the ordered *P4_3_32* structure lacks a plateau at 4.0 V due to its low Mn^3^⁺ content; instead, it exhibits a continuous long plateau at ≈4.7 V for the Ni^3^⁺/Ni^2^⁺ and Ni⁴⁺/Ni^3^⁺ redox reactions (Figure 2c).^[^ ^27^, ^35^ ^]^

The evolution of the two structural phases of LNMO during charging and discharging has been previously investigated.^[^ ^36^ ^]^ During Li delithiation, the lattice parameter of Li_x_Ni_0.5_Mn_1.5_O_4_ (*P4_3_32*) gradually decreased from 8.17 Å to 8.16 Å at x = 0.7, with the emergence of a second cubic phase structure starting to appear.^[^ ^30^, ^37^ ^]^ Notably, a third cubic phase forms when more than 50% of Li is removed. The lattice parameters of the three cubic phases were 8.17 Å, 8.08 Å, and 7.99 Å, respectively. The second cubic phase served acts as a buffer, coordinating between the first and third cubic phases.^[^ ^38^ ^]^ Conversely, for Li_x_Ni_0.5_Mn_1.5_O_4_ (*Fd3m*) (0.04 < x<1), the lattice parameter progressively decreased from 8.172 Å to 8.12 Å during Li deintercalation. This stage, characterized as a solid solution reaction, corresponded to the first voltage plateau in the charging curve.^[^ ^39^ ^]^ When the degree of deintercalation exceeded 50%, the structure began to transition gradually into a second cubic structure with the same space group.^[^ ^40^ ^]^ A third cubic structure emerged at x = 0.4, corresponding to the second voltage plateau in the charging curve. Importantly, the unit cell parameter decreased from 8.08 Å to 8.0 Å during this two‐phase reaction. Specifically, during charging, the *P4_3_32*‐type structure underwent a two‐phase reaction involving three distinct cubic structures: Li_1_Ni_0_._5_Mn_1_._5_O_4_ (Li_1_), Li_0_._5_Ni_0_._5_Mn_1_._5_O_4_ (Li_0_._5_), and Li_0_Ni_0_._5_Mn_1_._5_O_4_ (Li_0_).^[^ ^41^ ^]^ Conversely, the *Fd3m* structure exhibited no significant two‐phase reaction between Li_1_Ni_0_._5_Mn_1_._5_O_4_ and Li_0_._5_Ni_0_._5_Mn_1_._5_O_4_ but instead demonstrated a continuous phase‐transition process.

The LNMO octahedral 16c vacancy can accommodate Li^+^ at < 3.0 V.^[^ ^42^ ^]^ The structural changes in the charging and discharging phases of LNMO at < 3.0 V were previously investigated. Specifically, S.H. Park et al. compared the differences in voltage profiles between the two space group structures of LNMO over the 2.0–5.0 V voltage range.^[^ ^43^ ^]^ According to their findings, the voltage plateau of the *P4_3_32* exhibited no significant change during cycling, indicating that this structure could maintains good stability during Li (de) intercalation. In contrast, the *Fd3m* displayed a tetragonal phase at ≈3.0 V, which is attributed to the incomplete transformation of the tetragonal spinel phase into a cubic spinel phase following Li^+^ extraction.^[^ ^44^ ^]^

During charging and discharging, LNMO typically undergoes a two‐phase transition, which results in lattice volume changes.^[^ ^45^ ^]^ Such alterations induce substantial lattice strain and structural degradation in both lithium‐rich and lithium‐poor regions. During deep discharge below 3V, LNMO undergoes a complex cubic‐to‐tetragonal phase transition.^[^ ^46^ ^]^ In this process, Mn⁴⁺ is reduced to Mn^3^⁺, inducing structural distortion and the formation of the Li_2_Mn_2_O_4_ tetragonal phase. Concurrently, Ni ions migrate from the bulk phase to the surface, resulting in the formation of the rock‐salt phase.^[^ ^38^, ^47^ ^]^ The structural evolution during electrochemical cycling can be further revealed by advanced characterization techniques. Liu et al. revealed the phase transition mechanism of LNMO through full‐field transmission X‐ray microscopy technique.^[^ ^48^ ^]^ The study found that LNMO exhibits inhomogeneity and coexistence of lithium‐rich and lithium‐poor phases during the delithiation process, providing direct evidence for the phase transition mechanism. It was revealed that the phase transition does not follow the traditional core–shell reaction process or particle‐by‐particle reaction process. A recent study employing the in situ coherent X‐ray diffractive imaging technique showed that the strain energy is relatively low at the early stages of LMNO delithiation, but it increases by more than ten‐fold during the topotactic phase transformation.^[^ ^49^ ^]^ Jakub Drnec et al. further employed high‐energy microfocus X‐ray diffraction to investigate the state of LNMO in situ during charging and discharging,^[^ ^50^ ^]^ revealing a nonlinear coupling between the strain and the lithiation state of LNMO at high voltages, which suggests that the phase diagram of the material is more complex. Even at low currents, significant lithiation inhomogeneities form on the electrode cross‐section. Minggao Ouyang et al. utilized in situ synchrotron X‐ray diffraction and HRTEM to demonstrate that LNMO undergoes a high‐voltage two‐phase transition from Li_1−x_Ni_0_._5_Mn_1_._5_O_4_ to MnO_2_.^[^ ^51^ ^]^ This transition is associated with lattice strain accumulation and intra‐lattice cracking, ultimately leading to material degradation.

## 2 LNMO Structural Failure and Capacity Decay Mechanisms

### 2.1 Transition Metal Dissolution

**Figure 3:**
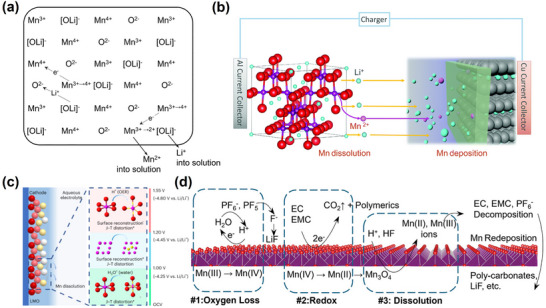
a) Schematic illustration of the conversion of LiMn_2_O_4_ to λ‐MnO_2_ in acidic aqueous solution. Reproduced with permission.^[^ ^52^ ^]^ Copyright 1981, Elsevier Inc. b) Schematic diagrams showing the dissolution, migration and deposition of manganese in lithium‐ion batteries. Reproduced with permission.^[^ ^69^ ^]^ Copyright 2016, WILEY‐VCH Verlag GmbH & Co. c) Mn dissolution mechanism in 2 M LiTFSI aqueous electrolytes. Reproduced with permission.^[^ ^71^ ^]^ Copyright 2023, Nature. d) Diagram of the manganese dissolution mechanism. Reproduced with permission.^[^ ^72^ ^]^ Copyright 2025, WILEY‐VCH Verlag GmbH & Co.

The disordered LiNi_0_._5_Mn_1_._5_O_4_₋δ has a Mn⁴⁺/Mn^3^⁺ redox pair. Therefore, the same transition metal dissolution phenomenon as in lithium manganate will occur. In 1981, Hunter reported the conversion of the spinel LiMn_2_O_4_ to λ‐MnO_2_ following treatment with an acidic aqueous solution.^[^ ^52^ ^]^ Although λ‐MnO_2_ retained the structural framework of LiMn_2_O_4_, some of the Li was removed from its tetrahedra, resulting in lattice contraction in the spinel structure. As Li⁺ and Mn^2^⁺ were removed from the surface, along with the conversion of Mn^3^⁺ to Mn⁴⁺, an increasing amount of Li⁺ diffused from the bulk to the surface. Notably, the researchers attributed the conversion reaction to a disproportionation reaction mechanism (**Figure** **3**a), which is described in Equation (1). Gummow et al. discovered that the concentration of manganese (IV) on spinel surfaces increased with dissolution.^[^ ^53^ ^]^ This mechanism was often consistent with experimental results, which revealed that manganese dissolution increased with an increase in acid concentration in the electrolyte, the specific surface area of active‐substance particles, and temperature.^[^ ^54^ ^]^

(1) $$2M{n^{3}}^{+}\to Mn^{2+}+Mn^{4+}$$

Oh and Nishikawa et al. further observed a divalent oxidation state of manganese in the electrolyte (Mn^2+^).^[^ ^55^ ^]^ However, the disproportionation reaction of Mn(III) could not explain the increased solubility of manganese at high charge potentials and the decreased Mn(III) concentration.^[^ ^56^ ^]^ Consequently, the researchers attributed the dissolution behavior of Mn at high charge potentials to other mechanisms, including phase transitions and chemical reactions (Figure 3c).^[^ ^57^ ^]^ This proposal could help resolve the Mn dissolution phenomenon in the spinel at the high‐potential plateau of the two‐phase coexistence. Notably, because of the temperature sensitivity of the two‐phase structure, the aforementioned reaction was accelerated at high temperatures.^[^ ^58^ ^]^ The concentration of these ions also increased with rising temperature.^[^ ^59^ ^]^ Xuejie Huang et al. also linked the accelerated Mn dissolution to the temperature sensitivity of the two‐phase structure.^[^ ^60^ ^]^ In this regard, the direct dissolution of Mn(iii) might also occur.

Consistent with the aforementioned findings, Arubach et al. also reported that Mn(iii), rather than Mn(ii), was the main soluble Mn ion in the electrolyte (Figure 3d).^[^ ^56^ ^]^ Furthermore, chemical lithiation and protonation during high‐temperature aging resulted in the formation of Mn‐deficient λ‐Li_x_H_y_MnO_2_ phases and the generation of Li‐rich spinel‐mediated protonated phases, implying that chemical lithiation and protonation could also lead to manganese solubilization.^[^ ^57^, ^58^ ^]^ In addition, electrolyte decomposition can lead to transition metal dissolution, as follows:

(2) $$\begin{array}{ccc} & & 16\mathrm{HF}+8\mathrm{LiN}i_{0.5}Mn_{1.5}O_{4}\to \\ & & 12Ni_{0.25}Mn_{0.75}O_{2}+\mathrm{Ni}F_{2}+3\mathrm{Mn}F_{2}+8\mathrm{LiF}+8H_{2}O \end{array}$$

The Mn dissolution mechanism during Li^+^ deintercalation/intercalation remains controversial. However, it is generally acknowledged that battery capacity degradation correlates directly with Mn dissolution.^[^ ^59^ ^]^ Capacity degradation has been established to directly cause the loss of the active material from the cathode material. However, material loss only accounts for 20–33% of total capacity loss in the battery.^[^ ^61^ ^]^ The large portion of Activated Charcoal (AC)impedance‐detected capacity loss of the material was attributed to increased electrode impedance. Specifically, Mn in the electrolyte, driven by a concentration gradient or electric field force, migrated to and deposited on the negative electrode, increasing negative electrode impedance.^[^ ^62^ ^]^ Amine et al. further reported that the impedance of the negative electrode (graphite) increased significantly when a small amount of Mn was present in the electrolyte, causing battery capacity degradation—a phenomenon that validates a previously mentioned observation.^[^ ^63^ ^]^ Commonly used negative electrodes (such as Li metal and graphite) have also demonstrated potentials below the redox potential of the Mn^2^⁺/Mn couple. To explain Mn deposition on the negative electrode, researchers proposed electrochemical or chemical reduction mechanisms,^[^ ^64^ ^]^ as shown in Equation (3):

(3) $$Mn^{2+}+2e\to \mathrm{Mn}$$

The reduction mechanism posits that the electrocatalytic properties of the Mn metal might exacerbate electrolyte decomposition.^[^ ^65^ ^]^ Therefore, battery capacity degradation could be attributed to the metal Mn deposition‐induced irreversible decomposition of the electrolyte.^[^ ^66^ ^]^ This irreversible decomposition hinders Li^+^ detachment/embedding and increases negative electrode impedance.^[^ ^67^ ^]^ Meanwhile, interfacial side reactions could consume Li^+^, leading to the loss of active Li, and potentially exacerbating capacity degradation. Delacourt et al. proposed the following manganese deposition process.^[^ ^68^ ^]^:

(4) $$Mn^{2+}+2e\to Mn^{0}$$

(5) $$Mn^{0}+\mathrm{EC}\to \mathrm{MnC}O_{3}+C_{2}H_{4}$$

Khalil Amine et al. proposed a Manganese Dissolution‐Migration‐Deposition (DMD) model to elucidate the manganese deposition process,^[^ ^69^ ^]^ as shown in Figure 3b. Through the ion exchange reaction with Li^+^ in the solid electrolyte interface (SEI), Mn^2+^ in the electrolyte accumulated in the negative electrode SEI, hindering Li^+^ diffusion and increasing negative electrode impedance. Furthermore, increasing temperatures enhanced the manganese DMD process, leading to poor material performance at high temperatures. Overall, manganese dissolution at the cathode electrode could lead to active material loss and increased impedance. On the other hand, manganese deposition at the negative electrode might cause capacity degradation via SEI membrane damage, hindering Li^+^ detachment/embedding and ultimately increasing negative electrode impedance.^[^ ^70^ ^]^

### 2.2 Jahn‐Teller Effect

**Figure 4:**
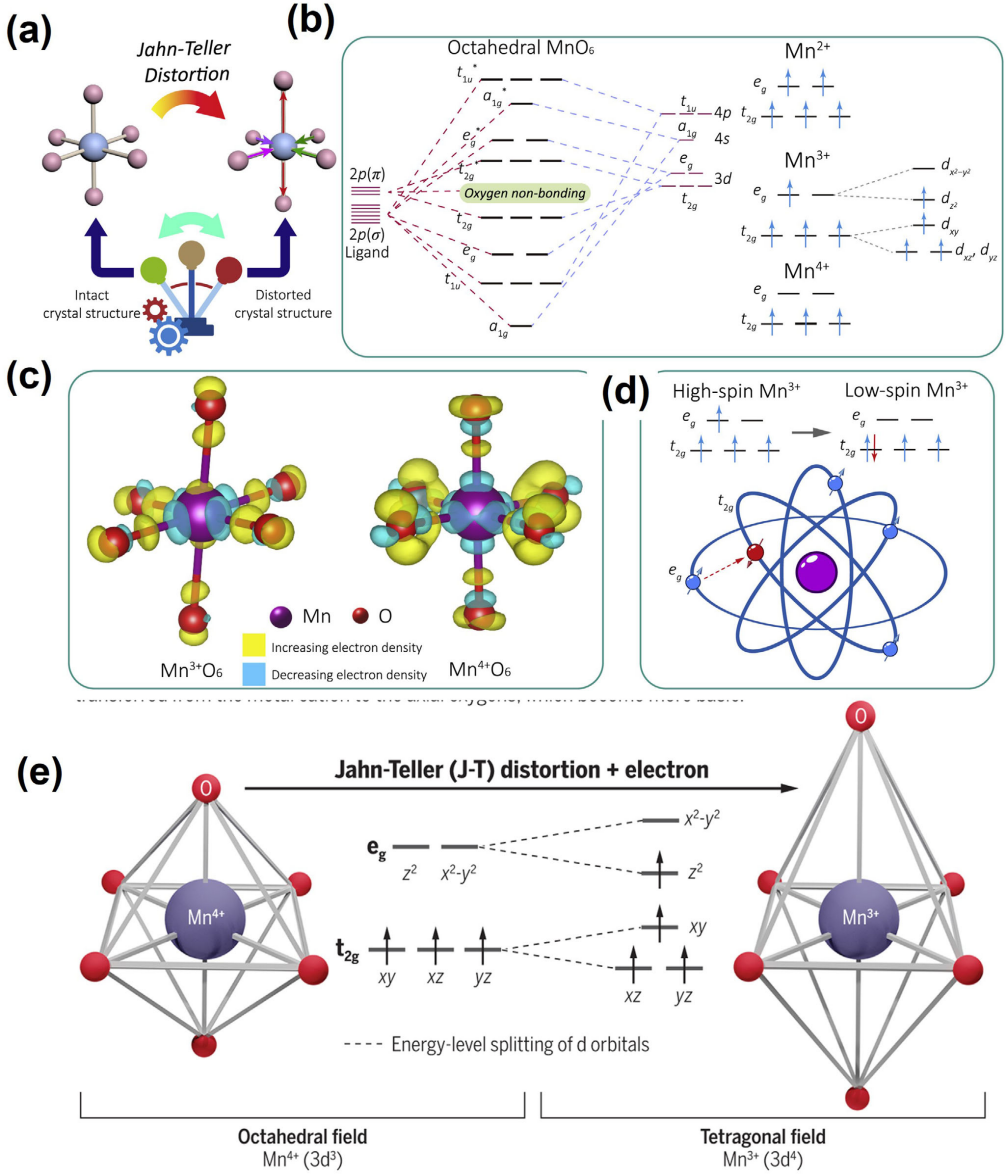
a) Schematic illustration of octahedral MnO_6_ before and after Jahn‐Teller distortion. Reproduced with permission.^[^ ^12^ ^]^ Copyright 2021, Elsevier Inc. b) The molecular orbital energy diagram of the octahedral MnO_6_ and the electronic orbitals of Mn^2+^/Mn^3+^/Mn^4+^ ions. Reproduced with permission.^[^ ^12^ ^]^ Copyright 2021, Elsevier Inc. c) Calculated differential charge densities of octahedral Mn^3+^O_6_ and Mn^4+^O_6_. Reproduced with permission.^[^ ^85^ ^]^ Copyright 2021, Nature. d) Schematic diagrams of Mn 3*d* orbitals with high‐/low‐spin Mn^3+^. Reproduced with permission.^[^ ^12^ ^]^ Copyright 2021, Elsevier Inc. e) The ligand field in manganese oxides used in lithium‐ion battery cathodes dis‐torts the six equivalent metal‐oxygen bonds (left) into two longer axial bonds and four shorter equatorial bonds (right). Reproduced with permission.^[^ ^80a^ ^]^ Copyright 2020, American Association for the Advancement of Science.

The Jahn‐Teller effect, first proposed by HA Jahn and E. Teller in 1937, posits that a nonlinear molecular system in an electronic degenerate state will be distorted, removing degeneracy, reducing symmetry, and ultimately lowering the total energy,^[^ ^73^ ^]^ as shown in **Figure** **4**a. A strong Jahn‐Teller effect typically occurs in TMs compounds when an odd number of electrons occupy TMs e_g_ orbitals (i.e., d_z_^2^ and d_x_^2^_‐y_^2^) of the corresponding octahedral (O_h_) ligand field.^[^ ^74^ ^]^ In an idealized octahedral (O_h_) ligand field, Mn^3+^ (high spin, t_2g_^3^e_g_^1^), Fe^4+^ (high spin, t_2g_^3^e_g_^1^), Ni^3+^ (low spin, t_2g_^6^e_g_^1^), and Cu^2+^ (t_2g_^6^e_g_^3^) are among the first‐row transition metal (TM) ions that exhibit strong Jahn‐Teller effects, primarily due to their eg‐band electron configurations.^[^ ^75^ ^]^ Manganese, one of the 3d TMs, has various oxidation states, and its electrochemical activity often corresponds to oxidation states between +2 and +4.^[^ ^12^, ^76^ ^]^ In the nonlinear field of the MnO₆ octahedron, Mn^3^⁺ is often in a high‐spin state with a very large magnetic moment. Furthermore, the cooperative Jahn‐Teller ordering of MnO_2_ plates along the elongated axes could result in a strong Jahn‐Teller distortion.^[^ ^77^ ^]^ Given their electronic structure, 3d TMs, including Mn, have 3d orbitals categorized into two groups: bis‐degenerate e_g_ orbitals (including d_x_^2^_‐y_^2^ and d_Z_^2^) and tri‐degenerate t_2g_ orbitals (including d_xy_, d_xz_, and d_yz_).^[^ ^12^, ^78^ ^]^ For high‐spin Mn^3+^ in octahedral coordination (t_2g_^3^e_g_^1^), only one electron occupies the e_g_ orbital (E_g_^1^), resulting in an asymmetric e_g_ orbital. Furthermore, electrostatic repulsion between the electrons in the d_z_^2^ orbital of the Mn ion and the non‐bonding electrons on the p‐orbitals of the ligand O ion stretches the O ion along the *z*‐axis, inducing a structural transition from a cubic to a tetragonal phase (Figure 4b,c).^[^ ^79^ ^]^

Unlike Mn^2+^ and Mn^4+^, Mn^3+^ has a unique electronic configuration featuring an e_g_ orbital with an unpaired electron. Notably, d orbitals of Mn^3+^ are divided into two groups: Triply degenerate t_2g_ orbitals and doubly degenerate e_g_ orbitals. Mn^3+^ in a high spin state has its electrons preferentially occupying the d_z_^2^ orbitals, resulting in an asymmetric electronic configuration and causing Mn‐O bond stretching. This asymmetry could lead to lattice distortion, reducing system energy and breaking electronic symmetry, further impacting material stability (Figure 4d).^[^ ^79^ ^]^ Compared to Mn^2+^ and Mn^4+^, Mn^3+^ also has a unique Jahn‐Teller effect. Orbital‐distributed electrons have different degrees of nucleus shielding across different directions. In the above structure, the d orbitals no longer conform to the O_h_ symmetry of the octahedron, causing central Mn^3+^ instability. To stabilize Mn^3+^, two of the longitudinal Mn─O bonds (*c*‐direction) within the octahedral MnO_6_ would have to be elongated, while the other four horizontal Mn─O bonds would be contracted.^[^ ^80^ ^]^ This distortion could reduce the MnO_6_ octahedron from an O_h_ symmetry to D_4h_, accompanied by degenerate orbital elimination, system energy reduction, and crystal structure distortion (Figure 4e).^[^ ^12^, ^81^ ^]^

The Jahn‐Teller effect has been established to significantly affect the diversity and stability of Mn‐based materials, especially in the A‐Mn‐O system (A = Li, Na, K), where different A‐Mn ratios and synthesis conditions could lead to structural variations.^[^ ^82^ ^]^ Specifically, the Jahn‐Teller effect could trigger mechanical stresses, leading to material fracture. According to research, phase transitions and structural distortions in the low‐pressure region could affect the cycling performance of batteries.^[^ ^83^ ^]^ The transition between the tetragonal and cubic phases could disrupt Li^+^ diffusion paths, resulting in slow kinetics and capacity degradation. Chemical reactions occurring in the Li─O─Li structure could also reduce the average valence state of Mn, with oxygen electrons lost for charge compensation—phenomena that further exacerbate the Jahn‐Teller effect.^[^ ^82^ ^]^ The Jahn‐Teller effect could induce transition metal ion migration, leading to voltage hysteresis and capacity decay. The phase transition further induces volumetric strain and crack formation, ultimately reducing the material's electrochemical reversibility. Therefore, eliminating the high spin state of Mn^3^⁺ could be key to improving the materials’ electrochemical performance.^[^ ^84^ ^]^

### 2.3 Electrolyte Decomposition

**Figure 5:**
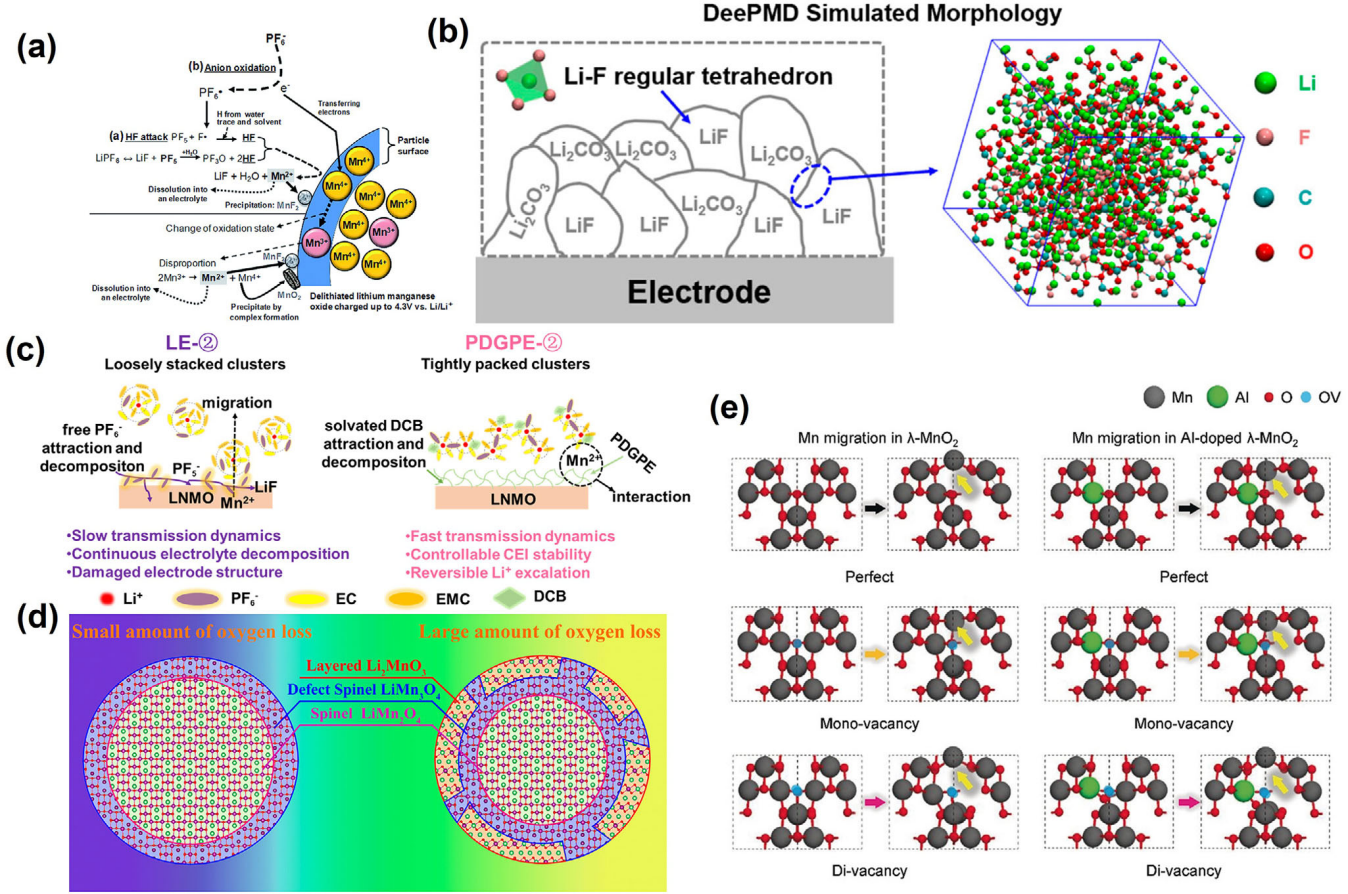
a) Schematic diagram of electrolyte decomposition. Reproduced with permission.^[^ ^95b^ ^]^ Copyright 2015, The Royal Society of Chemistry. b) Mosaic modeling of SEI on lithium battery electrodes. Reproduced with permission.^[^ ^91^ ^]^ Copyright 2022, Journal of the American Chemical Society. c) Schematic relationship between solvated structure and LNMO cathode stability in LE‐② and PDGPE‐② systems. Reproduced with permission.^[^ ^102^ ^]^ Copyright 2024, Elsevier Inc. d) Schematic diagram of manganese ion migration and structure formation on material surfaces. Reproduced with permission.^[^ ^103^ ^]^ Copyright 2017, American Chemical Society. e) Modeling of the migration paths of Mn ions in λ‐MnO_2_ and in structures with single and double vacancies. Reproduced with permission.^[^ ^104^ ^]^ Copyright 2019, Wiley‐VCH GmbH.

Besides the aforementioned mechanisms, electrolyte decomposition of LNMO at high voltages could also exacerbate its electrochemical performance degradation. Conventional Ethylene Carbonate (EC)‐based electrolytes have an upper electrochemical window of ~4.3 V and are likely to decompose at high voltages. For instance, LiPF_6_ decomposes to PF_5_, which further reacts with water remaining in the system to generate Hydrofluoric Acid (HF) (**Figure** **5**a).^[^ ^86^ ^]^ Furthermore, the generated HF somewhat corrodes the cathode material, causing capacity degradation.^[^ ^87^ ^]^ Notably, LNMO has a high charge/discharge voltage plateau (at 4.7 V), a charge cutoff potential (at ~5 V), and high particle activity at high voltages, leading to fast reaction speeds and severe interfacial side reactions, ultimately causing capacity degradation.^[^ ^88^ ^]^ During cycling, H⁺ and F⁻ ions dissociated from the electrolyte by‐product HF interact with reactive oxygen ions and transition metal ions, leading to the formation of H_2_O and fluoride compounds, respectively.^[^ ^89^ ^]^ Dissolved Mn^2^⁺ could exacerbate electrolyte decomposition, generating gases and reducing material safety. The trace water in the LiPF₆ electrolyte reacts with LiPF₆ through a series of processes outlined below (Equations 6–9).^[^ ^30^, ^90^ ^]^:

(6) $$\mathrm{LiP}F_{6}+H_{2}O\to \mathrm{LiF}+2\mathrm{HF}+\mathrm{PO}F_{3}$$

(7) $$\mathrm{PO}F_{3}+H_{2}O\to PO_{2}F_{2}H+\mathrm{HF}$$

(8) $$PO_{2}F_{2}H^{\cdot }+H_{2}O\to PO_{3}F{H_{2}}^{\cdot }+\mathrm{HF}$$

(9) $$PO_{3}F{H_{2}}^{\cdot }+H_{2}O\to PO_{4}H_{3}+\mathrm{HF}$$

During charging and discharging, with Li^+^ deintercalation/intercalation, more oxidizing nickel ions and manganese ions are gradually enriched on the electrode surface. At critical concentration values, these ions will promote electrolyte decomposition, generating inorganic (such as LiF) and organic (such as polycarbonate) substances, which are deposited at the interface of the material to form a cathode electrolyte interphase (CEI) film (Figure 5b).^[^ ^91^ ^]^ Although a proper CEI film can protect the cathode material and reduce its side reaction with the electrolyte, if too thick, it could hinder the transmission of Li^+^ and reduce their diffusion rate, leading to capacity degradation.^[^ ^92^ ^]^ The HF generated will not only dissolve the LiF in the CEI membrane and thus destabilize the CEI membrane, but also erode the LNMO, leading to the dissolution of the transition metal.^[^ ^93^ ^]^ The reaction formula is as follows:.^[^ ^90c^ ^]^

(10) $$\begin{array}{ccc} & & 16\mathrm{HF}+8\mathrm{LiN}i_{0.5}Mn_{1.5}O_{4}\to \\ & & 12Ni_{0.25}Mn_{0.75}O_{2}+\mathrm{Ni}F_{2}+3\mathrm{Mn}F_{2}+8\mathrm{LiF}+8H_{2}O \end{array}$$

The self‐discharge of LNMO in a charged state could also exacerbate electrolyte decomposition, illustrated as follows:.^[^ ^90c^ ^]^

(11) $$\mathrm{DEC}+\mathrm{LiP}F_{6}\to C_{2}H_{5}\mathrm{OCOOP}F_{4}+C_{2}H_{4}+\mathrm{HF}+\mathrm{LiF}$$

(12) $$CH_{5}\mathrm{OCOOP}F_{4}\to PF_{3}O+CO_{2}+C_{2}H_{4}+\mathrm{HF}$$

HF erodes the positive electrode, causing TMs dissolution. The dissolved TMs are further reduced and deposited to the negative electrode, increasing negative electrode impedance and restricting Li^+^ diffusion, ultimately resulting in capacity degradation.^[^ ^94^ ^]^ The precipitated Mn particles would cause SEI film thickening. Meanwhile, they would also consume active Li^+^, accelerating the full battery's capacity degradation.^[^ ^95^ ^]^

In summary, the influence of electrolyte on LNMO primarily manifests in three aspects: solvent, lithium salt, and additives. Complex interactions occur among solvents, lithium salts, and additives.^[^ ^96^ ^]^ Existing organic carbonate‐based electrolytes demonstrate relatively low stability (< 4.5 V vs. Li/Li⁺). Under high voltages, solvent molecules tend to undergo severe oxidative decomposition on the surface of the LNMO cathode, which not only corrodes LNMO but also induces transition metal dissolution, thereby resulting in battery capacity degradation.^[^ ^97^ ^]^ Common lithium salts such as LiPF₆ are susceptible to decomposition under high temperatures or voltages, producing corrosive substances like HF that attack LNMO and cause transition metal dissolution, thus affecting battery performance.^[^ ^98^ ^]^ However, the incorporation of specific additives can effectively mitigate these issues. Typical additives include those containing fluorine, phosphorus, nitrile groups, boron, silicon, and sulfur.^[^ ^99^ ^]^ These additives enhance the performance of LNMO batteries through multiple mechanisms, primarily by forming a stable cathode electrolyte interface (CEI) layer on the positive electrode and a solid electrolyte interface (SEI) layer on the negative electrode.^[^ ^100^ ^]^ These protective layers can effectively suppress lithium dendrite growth and electrolyte decomposition, stabilize electrode interfacial phases, and improve the cycling stability and overall performance of batteries.^[^ ^101^ ^]^

### 2.4 Oxygen Defects

During charging and discharging, excessive oxygen defects will damage the material's crystal structure, causing lattice distortion and structural collapse, thus reducing the material's specific capacity and shortening the battery's life cycle. Oxygen Vacancies (OVs), naturally occurring defects in TMs oxides, crucially influence their physicochemical properties. According to research, OVs in LIBs correlate closely with their capacity degradation mechanism.^[^ ^105^ ^]^ OVs ‐mediated capacity degradation is manifested in two key processes.^[^ ^106^ ^]^ First, OVs formation could lead to Mn ion migration (Figure 5d). Since the Mn migration barrier (≈0.2 eV) is smaller than the Li diffusion barrier (≈0.5 eV), Mn ion migration prevails. It is noteworthy that the migrated Mn ions are often unstable in OVs ‐containing tetrahedral sites, causing metal dissolution.^[^ ^104^ ^]^ Furthermore, excessive metal ion migration could extend to lattice planes, causing localized phase transitions, as well as lattice mismatches and microcracks, ultimately exacerbating electrolyte erosion and active material loss. After migration to empty octahedral sites, form rock salt phases (impurity phases), which hinder Li^+^ diffusion, leading to capacity decay and structural damage.^[^ ^103^ ^]^ The generation of microcracks could trigger degradation phenomena, including electrolyte decomposition and new solid electrolyte layer formation, potentially resulting in continuous capacity degradation and gas evolution. Moreover, the generation of gaps and microcracks could indirectly increase electrode material impedance.^[^ ^107^ ^]^ Second, the OVs formation process might cause manganese dissolution (Figure 5e). During OVs formation, charge replenishment occurs, yielding soluble Mn(II) and Mn₃O_4_, which are deposited on the negative electrode, increasing its impedance and decreasing Li^+^ diffusion.^[^ ^108^ ^]^ The oxidized organic electrolyte could decompose to form hydroxyl groups, which may react with surface Mn, leading to active material loss and capacity decay.^[^ ^109^ ^]^

The formation of oxygen vacancies in LNMO arises from the coupling of thermodynamic, kinetic, and electrochemical processes, and their behavior is intricately linked to the crystal structure, the redox activity of transition metals, and the interfacial chemistry of the electrolyte.^[^ ^110^ ^]^ For instance, during high‐temperature sintering (e.g., > 800°C), the thermal vibration of oxygen atoms intensifies, enabling some oxygen atoms to overcome lattice bonds and escape, thus forming oxygen vacancies.^[^ ^111^ ^]^ During charging and discharging, the induced Jahn‐Teller effect leads to an increase in local lattice stresses, potentially causing oxygen atoms to escape from the lattice. Additionally, electrolyte side reactions also promote the generation of oxygen vacancies. HF generated by the decomposition of LiPF₆ in the electrolyte attacks LNMO destabilizes the crystal lattice, and indirectly facilitates the formation of oxygen vacancies.^[^ ^112^ ^]^ Futhermore, the formation of oxygen vacancies varies significantly in distinct crystal structures (e.g., *Fd3m* and *P4_3_32*).^[^ ^27^, ^113^ ^]^ In the *Fd3m* structure, the formation energy of oxygen vacancies is higher, causing such vacancies to predominantly accumulate at the surface or grain boundaries rather than being uniformly distributed in the bulk phase. High concentrations of oxygen vacancies induce lattice remodeling (e.g., spinel → rock salt phase transition), which leads to rapid capacity decay. In contrast, the *P4_3_32* structure exhibits reduced symmetry, which reduce the migration barrier for oxygen atoms and results in a lower oxygen vacancy formation energy compared to *Fd3m*. These vacancies are more likely to aggregate at the crystal surface, promoting Mn migration and microcrack formation.

In conclusion, there is a close synergistic relationship among transition metal dissolution, the Jahn‐Teller effect, electrolyte decomposition, and oxygen vacancies in LNMO materials.^[^ ^114^ ^]^ For instance, the octahedral coordination environment of Mn^3^⁺ triggers the Jahn‐Teller effect due to asymmetric electron occupancy, causing tetragonal distortion of the crystal lattice. When exposed to protons, Jahn‐Teller effect serves as the active chemical driving force for spontaneous disproportionation, phase transformation, and transition‐metal dissolution.^[^ ^115^ ^]^ Oxygen vacancies adsorb polar molecules in the electrolyte and catalyze its decomposition, generating substances such as HF.^[^ ^116^ ^]^ HF reacts with the LNMO surface, dissolving transition metal ions (e.g., Mn and Ni), which further catalyze the decomposition of LiPF₆. Transition metal dissolution causes surface charge imbalance, triggering an irreversible spinel‐to‐rock salt phase transition.^[^ ^117^ ^]^ Meanwhile, structural collapse accelerates transition metal dissolution and interfacial side reactions, forming a vicious cycle.^[^ ^106b^ ^]^

## 3 LNMO Modification Research

Owing to its high‐voltage working platform, cost‐effectiveness, and eco‐friendliness, LNMO has a broad application prospect. However, it has also been associated with poor cycling stability and rate performance, limiting its application (especially at high temperatures, where TMs dissolution and surface side reactions exacerbate capacity decay). LNMO has recently been intensively investigated to enhance its electrochemical performance. The current modification strategies proposed for this purpose primarily include ion doping, surface modification, and morphology or crystal surface modulation, as summarized in **Table** **1**.^[^ ^13^, ^19^, ^97^, ^118^ ^]^

**Table 1:** Summary of modification strategies for LNMO.

Modification strategy	Advantages	Disadvantages
Doping modification	Enhance structural stability Regulate the valence state of transition metals Inhibit irreversible phase transition	Decrease in initial capacity Formation of inactive phases Increase in interfacial impedance
Surface coating	Isolates electrolyte erosion Regulate CEI/SEI films Buffer volume changes	Increase in ionic transport barrier Difficult to coat uniformly Low interfacial compatibility
Morphology and size management	Improve ionic/electronic transport efficiency Optimize structural stability Enhance interfacial contact	Acceleration of surface side reactions Complex preparation process Decrease in structural strength
Surface orientation management	Optimize electrochemical activity Improve structural stability Regulate lithium‐ion diffusion kinetics	Complex preparation process Contradiction in selective crystal facet exposure Unclear mechanism of action

### 3.1 Doping Modification

Elemental doping has recently been pervasively employed to improve materials’ electrochemical properties. According to research, elemental doping can adjust the electronic configuration and lattice parameters of Mn‐O‐Sub, optimizing the Li^+^ diffusion pathway.^[^ ^119^ ^]^ Moreover, cation disordering can effectively suppress the Jahn‐Teller distortion of Mn₃⁺O₆ octahedra.^[^ ^120^ ^]^ The ionic radius of the dopant element significantly influences the material's lattice parameter.^[^ ^121^ ^]^ It has also been established that smaller radius element doping shrinks the lattice, producing the microstrain and strengthening the Mn─O bond, ultimately enhancing the material's structural stability. On the other hand, larger radius elements could expand the lattice parameters, promoting Li^+^ diffusion and improving rate performance.^[^ ^122^ ^]^ Furthermore, substituting elements at different sites could yield different modification effects, as well as improve the structural stability and electrochemical properties of LNMO. Presently, doping avenues are mainly categorized into three groups: metal cation doping, nonmetal ion doping, and multi‐element co‐doping.

**Figure 6:**
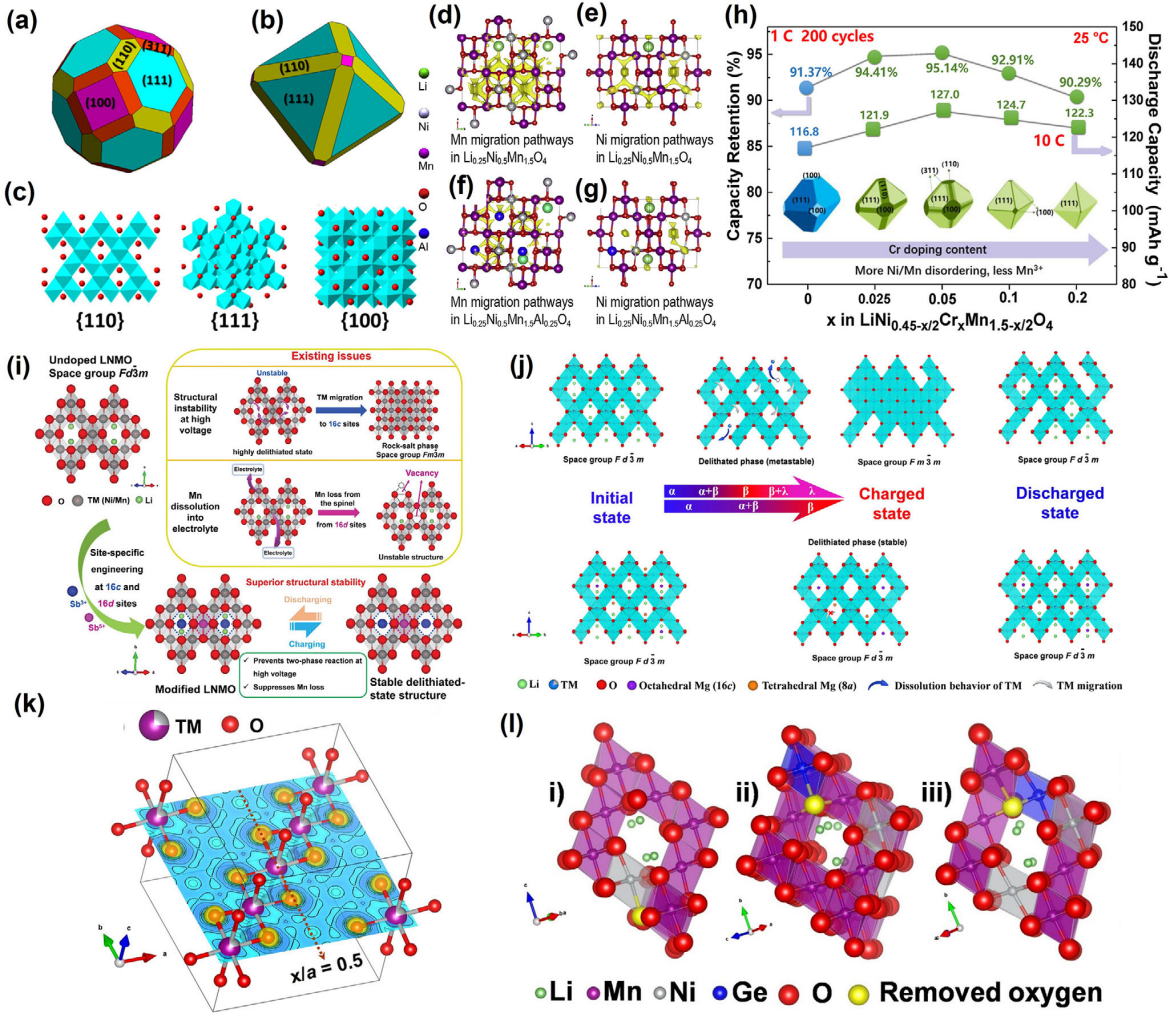
Schematic illustration of the Wulff shape of a) undoped and b) WO_3_‐doped LiMn_1.5_Ni_0.5_O_4_, c) and lithium‐ion diffusion channels on the (110), (111) and (100) faces. Reproduced with permission.^[^ ^125^ ^]^ Copyright 2017, American Chemical Society. d–g) Theoretical calculations regarding the transition metal (TM) ion mobilization in LNMO samples were carried out. The TM ion mobilization paths were investigated as follows: d) the mobilization path of Mn in Li_0.25_Ni_0.5_Mn_1.5_O_4_, e) the mobilization path of Ni in Li_0.25_Ni_0.5_Mn_1.5_O_4_, f) the mobilization path of Mn in Li_0.25_Ni_0.5_Mn_1.5_Al_0.25_O_4_, and g) the mobilization path of Ni in Li_0.25_Ni_0.5_Mn_1.5_Al_0.25_O_4_. All these calculations were based on the BV force field method. Reproduced with permission.^[^ ^126^ ^]^ Copyright 2018, Elsevier Inc. h) Conformational evolution of Cr^3+^‐doped LiNi_0.5‐x/2_Cr_x_Mn_1.5‐x/2_O_4_ samples. Reproduced with permission.^[^ ^129^ ^]^ Copyright 2021, Elsevier Inc. i) Schematic crystal structure of undoped LNMO and schematic structure and mechanism of site‐specific doped LNMO. Reproduced with permission.^[^ ^131^ ^]^ Copyright 2021, Wiley‐VCH GmbH. j) Schematic display of the structural changes and phase evolution of undoped LNMO and Mg0.1‐LNMO during charging and discharging. Reproduced with permission.^[^ ^132^ ^]^ Copyright 2020, Wiley‐VCH GmbH. k) Kernel density contour plots of LNMO and schematic diagrams of the Ef(vac) calculated crystal structures of l) LNMO (i) and doped Ge occupying (ii) 16c and iii) 16d. Reproduced with permission.^[^ ^130^ ^]^ Copyright 2022, Wiley‐VCH GmbH.

Doping with various metal elements mainly includes Na⁺, Mg^2^⁺, Sr^2^⁺, Al^3^⁺, Ga^3^⁺, Ge^3^⁺, Sb^3^⁺ (or Sb⁵⁺), Sc^3^⁺, Ti⁴⁺, V⁵⁺, Cr^3^⁺, Fe^3^⁺, Co^3^⁺, Cu^2^⁺, and Zn^2^⁺.^[^ ^123^ ^]^ Fuzhong Wu et al. introduced Sr to enhance the exposure of (100) crystalline facets, transforming the material morphology from octahedral to truncated octahedral with exposed (100) facets.^[^ ^124^ ^]^ The exposed (100) crystalline surface effectively suppressed the electrolyte side‐reaction and facilitated Li⁺ extraction/insertion. The Sr‐doped material also demonstrated excellent electrochemical performance, with a capacity retention of 86.63% after 500 cycles at 1C. Jian Luo et al. further increased the (110) and (111) crystal surface area via W anisotropic surface segregation, improving the Li^+^ diffusion rate and enhancing rate performance,^[^ ^125^ ^]^ as shown in **Figure** **6**a–c. The surface segregation and partial reduction of W further inhibited the formation of surface Mn^3^⁺, improving CEI film and cycling stabilities. On the other hand, LiJun Wan et al. introduced Al^3^⁺ on the 16c vacancies of octahedra, increasing surface stability, and thus suppressing structural decay during cycling (Figure 6d–g).^[^ ^126^ ^]^ Introducing Al^3^⁺ at the 16c site also inhibited the dissolution of TMs ions, promoted Li⁺ diffusion, inhibited electrolytic interfacial side reactions, and improved the cycling and rate properties of the materials. Given that Cr, Fe, and Co have ionic radii comparable to that of Mn, their Ni doping could help avoid the distortion of the LNMO crystal structure. Xiaogang Zhang et al introduced Cr into LiNi_0_._5_Mn_1_._5_O_4_ and found that it could eliminate the LiNi_1‐y_O impurity phase, decelerate transition metal dissolution, and improve both ionic and electronic conductivity.^[^ ^127^ ^]^ Furthermore, Lingxiao Lan et al.^[^ ^128^ ^]^ reported that introducing Sr^2+^ could reduce Mn ions, increase the Li─O bond strength, decrease the Li⁺ diffusion energy barrier, improve the thermodynamic stability of Li^+^ deintercalation/intercalation, and reduce the material's structural strain. Nonetheless, Li Wang et al. reported that Cr^3^⁺ increased the Ni/Mn disorder and reduced the Mn^3^⁺ content.^[^ ^129^ ^]^ Additionally, the material's morphology changed with the increasing doping amount (x) of Cr^3^⁺ (Figure 6h). At x ≤ 0.05, the (110) and (311) crystal planes with higher surface energy were exposed successively. At x = 0.1 and 0.2, the (311) crystal planes disappeared, the (100) and (110) planes decreased, and the (111) planes gradually increased. Overall, introducing Cr^3^⁺ eliminated the Li_x_Ni_1−x_O impurity phase, enhanced the degree of the Ni/Mn disorder, lowered Mn^3^⁺ content, and enhanced the material's structural stability due to the Cr─O bond. Furthermore, it lowered the proportion of (111) crystal planes and enhanced the exposure of the (100), (110), and (311) crystal planes, endowing the modified materials with excellent rate performance and cycling stability.

The decay in the structural stability of materials has been linked to weak orbital interactions (3d‐2p) between TMs and oxygen. Using LiNi_0_._5_Mn_1_._5_O_4_ as an example, Zaiping Guo et al. introduced strong 4s‐2p orbital hybridization to construct a robust TM‐O framework.^[^ ^130^ ^]^ (Figure 6k,l). According to the results, Ge2s‐O2p orbital hybridization increased the formation energy of OV defects and strengthened the TM‐O bonds, maintaining the material's structural stability. Furthermore, the capacity retention rate reached 71.4% after 2000 cycles at 1C. On the other hand, Wei Kong Pang's team prepared the LiNi_0.5_Mn_1.48_Sb_0.02_O_4_ material using the high‐temperature solid‐state method.^[^ ^131^ ^]^ (Figure 6i). They found that introducing the Sb element occupied the 16c site of the lattice, effectively enhancing the Mn─O bonding energy and exerting a structural pillar effect. Furthermore, the 16d site Sb enhanced the Ni/Mn disorder, stabilized the Mn─O bond, inhibited TM dissolution, and provided up to 99% of the theoretical specific capacity and energy density at the 1C rate. Additionally, Vanessa K. Peterson et al.^[^ ^132^ ^]^ introduced Mg into the tetrahedral (8a) and octahedral (16c) sites to inhibit the two‐phase reaction, enhance the material's structural stability, and suppress the Jahn‐Teller distortion effect (Figure 6j). The modified material exhibited capacity retention values of 86.3% and 87.3% after 1500 cycles at 1C and 2200 cycles at 10C, respectively. Yongyao Xia et al.^[^ ^133^ ^]^ also introduced Nb to limit the growth of the (111) crystal plane and transformed the material from an octahedral to a spherical structure with a small surface area via Nb catalytic action, which suppressed Mn dissolution and reduced the contact area with electrolytes to inhibit side reactions. They achieved an improved electrochemical performance, and the capacity retention rate was 74.1% after 500 cycles at 1C.

**Figure 7:**
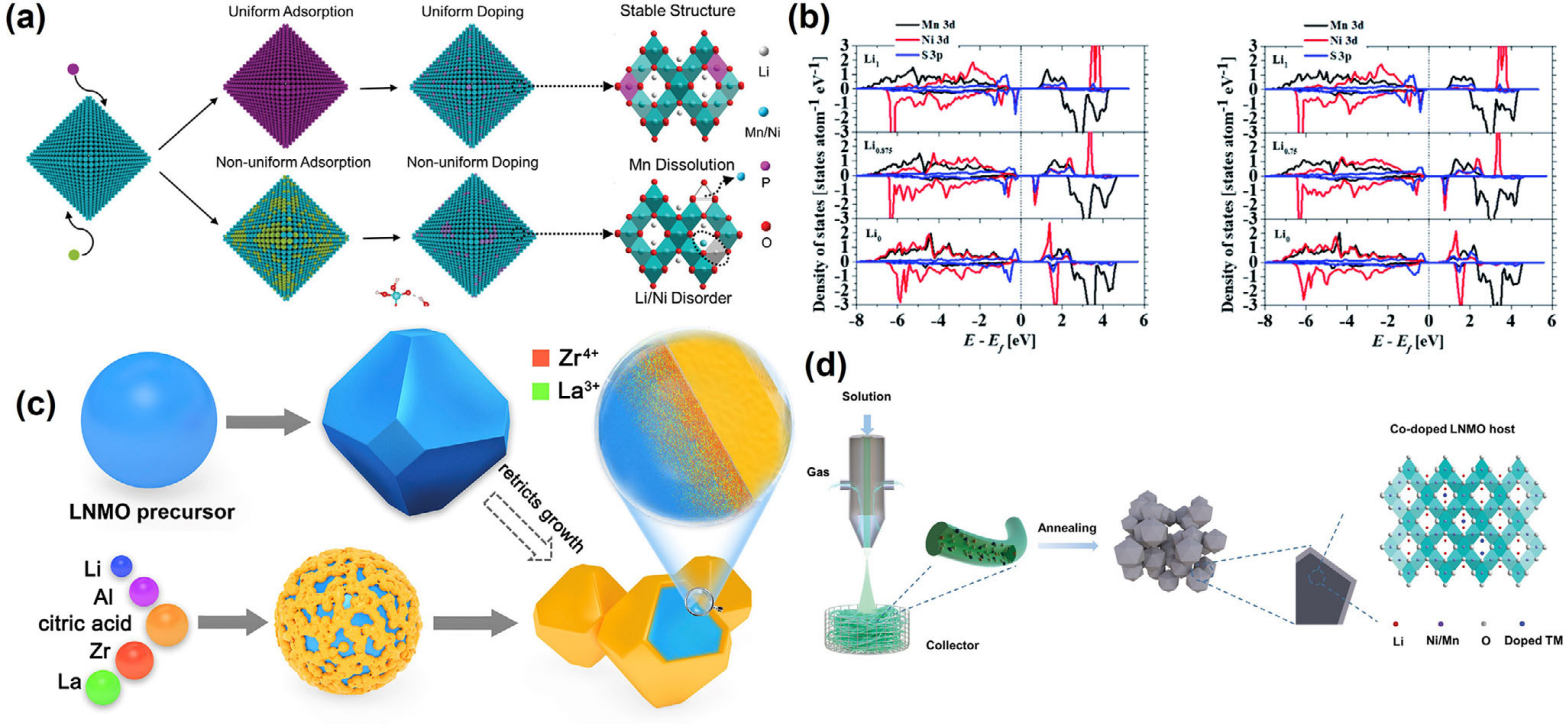
a) Schematic representation of the effect of uniform and nonuniform doping of P on the crystal structure of LNMO. Reproduced with permission.^[^ ^136^ ^]^ Copyright 2024, Wiley‐VCH GmbH. b) *x* = 0.125 and *x* = 0.25: DFT calculations on the changes in the partial density of states (PDOS) for Ni‐3d, Mn‐3d, and S‐3p orbitals. Reproduced with permission.^[^ ^138^ ^]^ Copyright 2020, The Royal Society of Chemistry. c) Schematic representation of LLAZO‐modified LNMO cathode materials. Reproduced with permission.^[^ ^140^ ^]^ Copyright 2020, Elsevier Inc. d) Schematic diagram of Cu, Fe, and Cr co‐doped LNMO prepared by electrostatic spinning. Reproduced with permission.^[^ ^141^ ^]^ Copyright 2022, Wiley‐VCH GmbH.

During material synthesis, LNMO generates OVs for charge replenishment, causing the electrochemical properties of the material to decay.^[^ ^134^ ^]^ Therefore, the doping of nonmetallic elements could effectively inhibit OVs formation in LNMO, improving the material's electrochemical properties. Presently, doping with nonmetallic elements primarily includes F⁻, Cl⁻, S^2^⁻, B^3^⁺, and P^3^⁺, among others.^[^ ^2^, ^135^ ^]^ Besides suppressing OVs, this doping approach could also enhance the material's electrochemical performance in multiple aspects, including improving its cycling stability and rate capability. Weiwei Sun et al. examined the effect of P doping on the interaction between TMs Mn, Ni, and O,^[^ ^136^ ^]^ as shown in **Figure** **7**a. According to the results, P doping significantly enhanced the interaction between Mn 3d‐O2p and Ni 3d‐O2p orbitals and effectively inhibited the Jahn‐Teller effect and Mn dissolution. It also reduced the energy barrier of Li^+^ de‐embedding kinetics, with a capacity retention of 97.4% after 100 cycles, which is significantly higher than that of unmodified materials. Furthermore, Jingying Xie prepared fluorine‐doped LiNi_0_._5_Mn_1_._5_O_4_ using NH_4_F as the fluorine source.^[^ ^137^ ^]^ Fluorine introduction transformed the material structure from an ordered *P4_3_32* to a disordered *Fd3m*. It also suppressed the interfacial side reaction between the electrolyte and the material, reducing material impedance; hence, the material showed better long‐term cycling stability and rate performance. On the other hand, Katsuya Teshima et al.^[^ ^138^ ^]^ reported that dope S^2^⁻ occupied the O24e site (*P4_3_32*), stabilized the Ni^2^⁺ and Mn⁴⁺ bonding energies, moderated Li⁺/Ni^2^⁺ cation mixing, and reduced the surface energy of the (100) crystal surface. They also found that the S‐3p orbitals exhibited higher energy levels than the Ni‐3d orbitals (Figure 7b), narrowing the band gap and lowering the Ni^2^⁺/Ni^3^⁺ redox overpotential, which, in turn, collectively enhanced the material's specific capacity and cyclic stability.

Multi‐element co‐doping has been established to play a multifunctional role through its synergistic effect. It could inhibit the degradation of LNMO materials and improve their electrochemical properties. Furthermore, it could enhance the materials’ structural stability and electrochemical properties. Nobuyuki Zettsu et al.^[^ ^139^ ^]^ found that Cu^2^⁺/F⁻ double doping altered the electronic structure of the materials, reduced the activation energy of Li⁺ migration to octahedral vacancies, and mitigated the lattice distortion resulting from the insertion of additional Li⁺ into the spinel framework. Consequently, it suppressed the irreversible phase transition from the cubic to the tetragonal phase and yielded a high specific capacity, good rate performance, and excellent cycling stability. Furthermore, Ying Bai et al. introduced La^3+^ and Zr^4+^ into the LNMO lattice, creating a Li‐La‐Zr‐O transition layer that shortened the Li^+^ diffusion path (Figure 7c).^[^ ^140^ ^]^ Compared to the pristine sample, the modified sample showed better cycling stability, with a capacity retention rate of 72.6% after 600 cycles. Additionally, Hong‐Bin Yao et al.^[^ ^141^ ^]^ synergistically modified LNMO through Cr‐Fe‐Cu multi‐ion co‐doping (Figure 7d) to stabilize the LNMO matrix, inhibit Mn dissolution, and improve Li⁺ ion diffusion and electronic conductivity. According to the results, LNMO achieved long‐term cycling stability, with 78% capacity retention after 300 cycles at 1C. Doping strategies for modifying LNMO are summarized in **Table** **2**.

**Table 2:** Summary of doping strategies to modify LNMO.

Doping element	Doping method	Cycling performance	Rate capability	Ref.
Cr	Sol–gel	116.2 mAh g^−1^ after 1000 cycles at 1C (capacity retention 82.75%)	92 mAh g^−1^ at 5C	[127]
Zr	Low temperature solution combustion	138.4 mAh g^−1^ after 200 cycles at 1C (capacity retention 95.0%)	129.4 mAh g^−1^ at 10C	[142]
B	Solid‐state	130.3 mAh g^−1^ after 500 cycles at 1C (capacity retention 86.3%)	104.5 mAh g^−1^ at 7C	[135]
Sr	Sol–gel	134.6 mAh g^−1^ after 500 cycles at 1C (capacity retention 86.63%)	109.6 mAh g^−1^ at 20C	[124]
Ti	Spray drying	139 mAh g^−1^ after 500 cycles at 0.5C (capacity retention 95.6%)	105.8 mAh g^−1^ at 5C	[143]
Sc	Solution combustion	102 mAh g^−1^ after 1000 cycles at 5C (capacity retention 98%)	80 mAh g^−1^ at 12C	[144]
V	Sol–gel	130 mAh g^−1^ after 100 cycles at 1C (capacity retention 94%)	58 mAh g^−1^ at 5C	[145]
Fe	Coprecipitation	111.8 mAh g^−1^after 300 cycles at C/3 (capacity retention 90.2%)	108 mAh g^−1^ at 5C	[146]
F	Solid state	115.2 mAh g^−1^ after 300 cycles at 1C (capacity retention 92.4%)	105 mAh g^−1^ at 10C	[137]
Al–F	Solid state	124 mAh g^−1^ after 200 cycles at 1C (at 55 °C, capacity retention 92%)	118.4 mAh g^−1^ at 10C	[147]
Mg–Cr	Coprecipitation	105.6 mAh g^−1^ after 200 cycles at 5C (capacity retention 95.4%)	110.7 mAh g^−1^ at 10C	[123d]
Cr‐Fe‐Cu	Blow‐spinning	78% capacity retention after 300 cycles at 1C (full‐cell)	3 mAh cm^−2^ at 0.5C	[141]

Elemental doping can strengthen the material's crystal structure without changing the spinel skeleton. This phenomenon might effectively suppress the material's structural collapse due to the phase transition during charging and discharging. It could also lower the Li⁺ transport barrier and shorten the Li⁺ transport channel, thus accelerating the Li⁺ transport rate. Doping elements on the surface could also create a stable material interface, suppressing the manganese dissolution‐induced capacity loss. Various doping element valences and ionic radii have been utilized to achieve substitution in the LNMO lattice across different sites. It is also noteworthy that high‐entropy doping has been widely employed in LIBs. It could strengthen the lattice framework to form a stable crystal structure and suppress Li⁺ deintercalation‐induced volumetric strain. Furthermore, multi‐element doping could change the material's electron cloud distribution, optimize the electronic structure, and endow the material with a more excellent energy band structure and electronic density of states. This phenomenon allows for the transport and transfer of electrons, improving the battery's electrochemical performance.

### 3.2 Surface Coating

Surface coating is primarily aimed at resolving the degradation of battery performance resulting from electrolyte decomposition and interfacial side reactions of cathode materials during charging and discharging.^[^ ^148^ ^]^ According to research, surface coating can effectively suppress surface side reaction and manganese dissolution, as well as improve the battery's electrochemical performance.^[^ ^97^, ^149^ ^]^ These outcomes could be achieved through five key avenues. First, a physical barrier could be created to block side reactions between the cathode material and electrolyte, especially under high‐temperature and high‐pressure conditions. During charging and discharging, the barrier could effectively prevent direct contact between the cathode material and the electrolyte, limiting the electrolyte‐mediated erosion of the cathode material. Second, an HF scavenger could be leveraged to prevent the chemical erosion of the nonaqueous electrolyte and active substance loss. Third, an electron‐ and ion‐conducting layer could be introduced to enhance the charge transfer at the cathode/electrolyte interface and reduce the charge‐transfer resistance (R_ct_). Fourth, a chemical surface modifier could be introduced to promote ionic charge transfer at the anode/electrolyte interface. Notably, the coating material could also diffuse to the particle surface to change the cathode material's surface chemistry, creating a new interface layer that enhances structural stability and charge/ion transfer. Finally, interfacial stability could be enhanced to reduce the volumetric strain during charging/discharging. The coating materials presently being used pervasively include metal oxides, metal fluorides, phosphates, polymers, Li conductive coatings, and electronic conductive coatings, among others.

**Figure 8:**
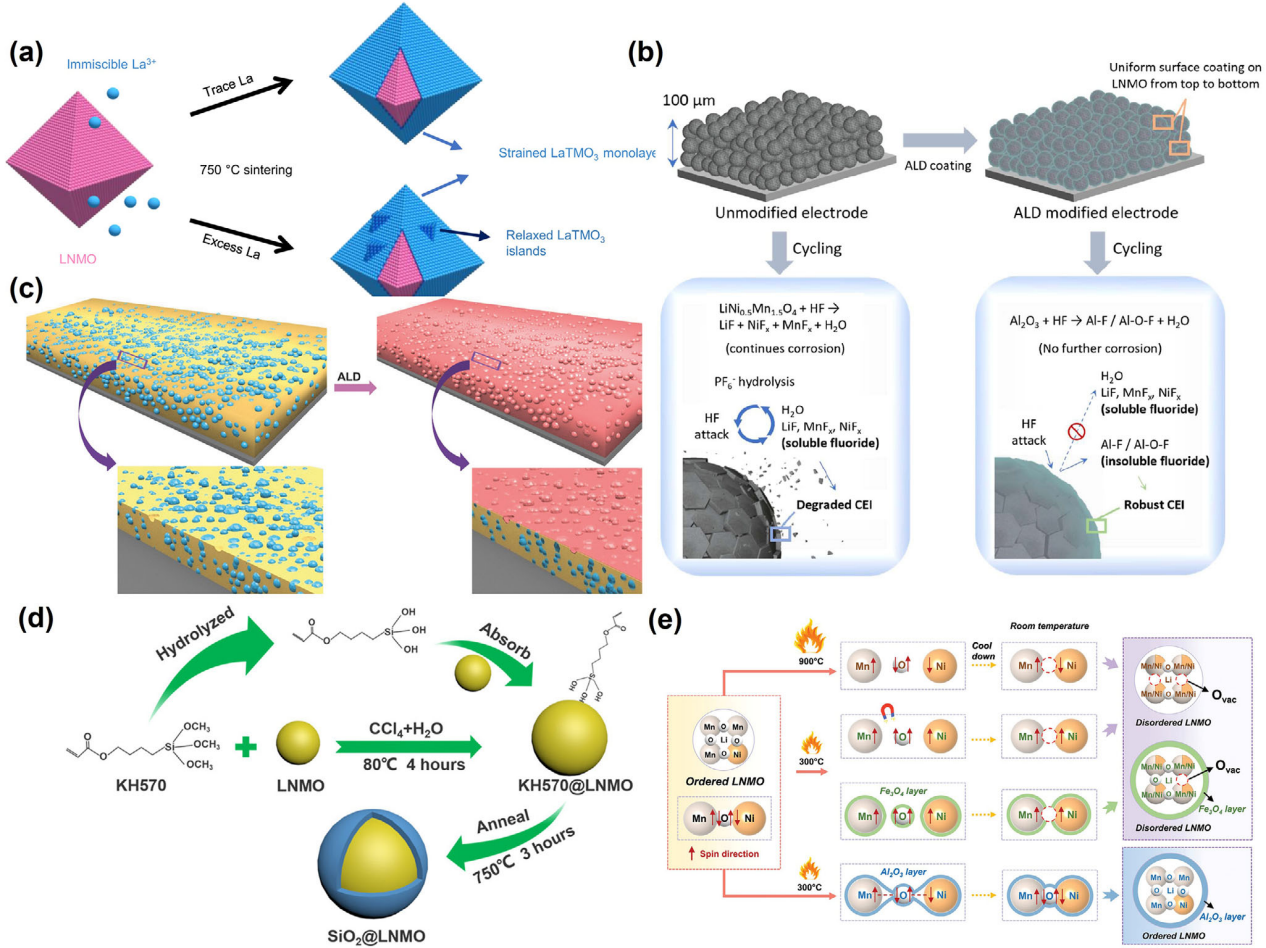
a) Schematic of LaTMO_3_ growth on LNMO surface. Reproduced with permission.^[^ ^151^ ^]^ Copyright 2022, Nature. b) Schematic diagram of ALD improved LNMO electrode performance. Reproduced with permission.^[^ ^153^ ^]^ Copyright 2022, Elsevier Inc. c) Schematic diagram showing ALD on electrodes. Reproduced with permission.^[^ ^154^ ^]^ Copyright 2017, Wiley‐VCH GmbH. d) Preparation process for siloxane surface‐modified LNMO. Reproduced with permission.^[^ ^155^ ^]^ Copyright 2021, American Chemical Society. e) Schematic representation of the phase transition mechanism in different cases of Fe_2_O_3_ and Al_2_O_3_. Reproduced with permission.^[^ ^152^ ^]^ Copyright 2024, Wiley‐VCH GmbH.

Metal oxides serve as a physical barrier between the cathode material and the electrolyte, effectively improving the material's thermal and long‐cycle stabilities. Some of the commonly used metal oxides include Al_2_O₃, TiO_2_, ZrO_2_, V_2_O_5_, La_2_O₃, Ce_2_O₃, and MgO.^[^ ^150^ ^]^ Lianzhou Wang et al. constructed a LaTMO_3_ (TM = Ni, Mn) passivation layer at the LiNi_0.5_Mn_1.5_O_4_ interface using an epitaxial strategy (**Figure** **8**a).^[^ ^151^ ^]^ They found that the LaTMO_3_ layer had a thin atomic structure, which correlated with the LiNi_0.5_Mn_1.5_O_4_ lattice and blocked TM dissolution. The epitaxial strategy effectively improved the cycling stability of LiNi_0.5_Mn_1.5_O_4_, with the capacity maintained at ~77% after 1000 cycles at 290 mA g^−1^. Furthermore, Xiaoning Li et al. induced phase transitions at lower temperatures using a magnetic Fe_3_O_4_ shell‐generated localized magnetic field (Figure 8e).^[^ ^152^ ^]^ The magnetic field initiated a free radical pairing mechanism and induced the Ni/Mn ordered–disordered phase transition even at lower temperatures. The electrochemical analysis also revealed that the enhanced Ni/Mn disordered structure significantly improved the electrode's cycling and rate performances. Additionally, Ying Shirley Meng et al. introduced an Al_2_O_3_ coating to the LNMO interface through Atomic Layer Deposition (ALD) (Figure 8b).^[^ ^153^ ^]^ The Al_2_O_3_ coating was converted to Al–O /Al–F during cycling, improving the interfacial stability and protecting the cathode material. Meanwhile, the cladding layer inhibited the HF mediated erosion. Chongwu Zhou et al. further examined the mechanism of the Al_2_O₃ coating in effectively suppressing Mn^2^⁺ formation on the electrode surface and manganese dissolution (Figure 8c).^[^ ^154^ ^]^ The coating blocked the direct contact between the material and the electrolyte, reducing the impedance and alleviating capacity degradation. Gianluigi A. Botton et al. also introduced a TiO_2_ coating on the LiNi_0.5_Mn_1.5_O_4_ surface via ALD.^[^ ^150d^ ^]^ The TiO_2_ coating suppressed the formation of impurity phases such as Li_x_Ni_1‐x_O and electrolyte erosion. Some titanium atoms further diffused into the material to occupy the 8a tetrahedral sites, creating a cation‐deficient spinel phase similar to TiMn_2_O_4_ (TMO), thus contributing to homogeneous SEI formation. It was also reported that more titanium doped into the octahedral sites suppressed the surface phase transition and improved the material's structural stability, Coulombic efficiency, and rate performance. Finally, Guangshi Tang et al.^[^ ^155^ ^]^ leveraged the easy hydrolysis and adsorption properties of γ‐methyl‐propylene trimethoxysilane (KH570) for the dual modification of Si doping and SiO_2_ coating (Figure 8d). This approach improved the kinetics of the material without affecting the electrochemical activity of the material interface. Furthermore, the capacity retention rates were 88% after 800 cycles at 1C and 89.7% after 1000 cycles at 3C. The material also retained its hydrolytic and adsorptive properties.

**Figure 9:**
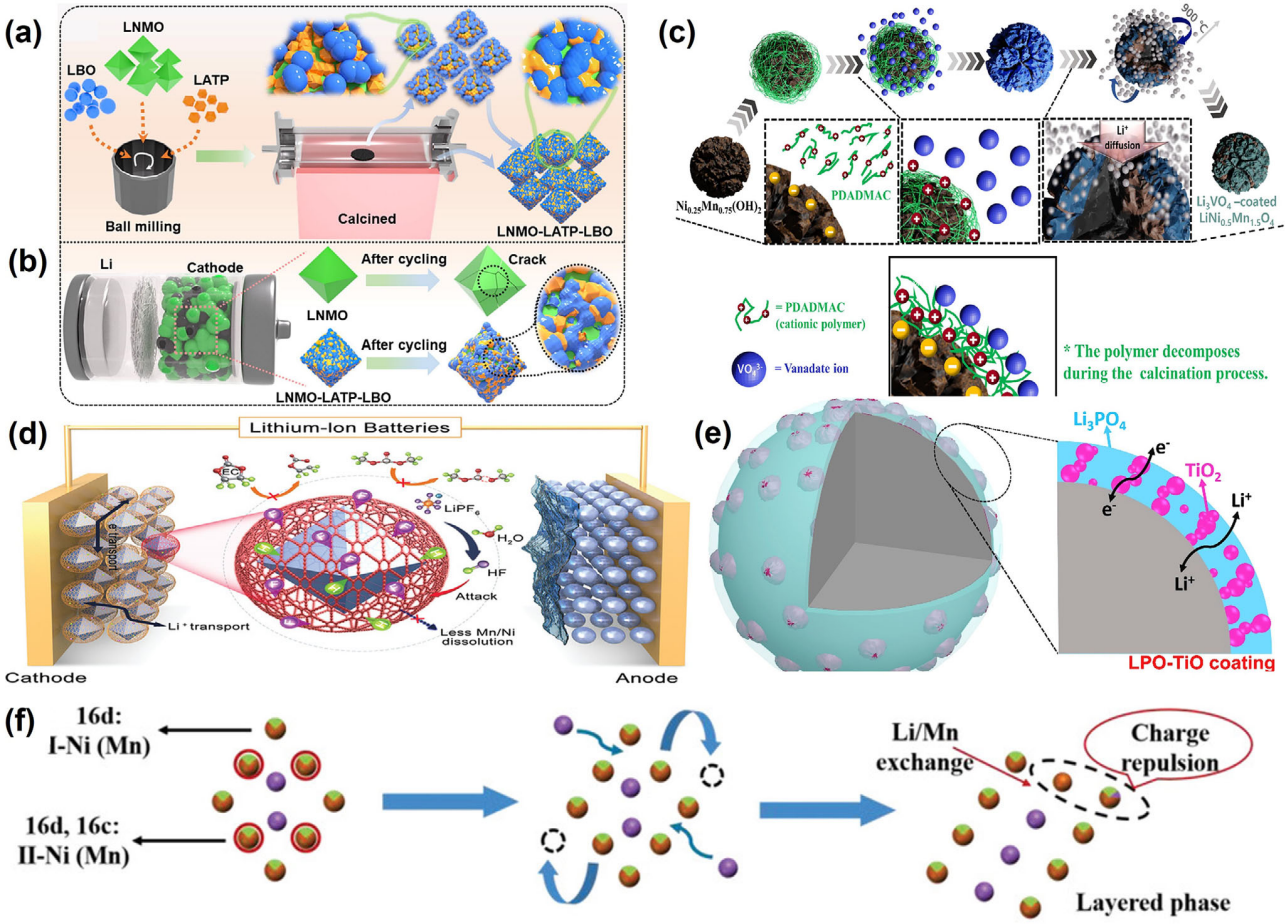
a) Schematic display of LNMO‐LATP‐LBO synthesis and b) Schematic representation of LNMO and modified LNMO in the initial state and after cycling. Reproduced with permission.^[^ ^156^ ^]^ Copyright 2022, Elsevier Inc. c) Schematic illustration of the synthesis of Li₃VO_4_‐coated LNMO. Reproduced with permission.^[^ ^157^ ^]^ Copyright 2022, Elsevier Inc. d) Schematic diagram showing the LNMO protected by GDY cladding. Reproduced with permission.^[^ ^162^ ^]^ Copyright 2021, Wiley‐VCH GmbH. e) Schematic structure of LPO‐TiO‐coated LNMO. Reproduced with permission.^[^ ^167^ ^]^ Copyright 2019, Elsevier Inc. f) Schematic diagram of the phase transition at the interface of HT‐LNMO‐150. Reproduced with permission.^[^ ^168^ ^]^ Copyright 2022, Wiley‐VCH GmbH.

On the other hand, Li‐conducting coatings can improve the Li transfer rate and effectively enhance LNMO electrochemical performance. Ying Bai et al.^[^ ^140^ ^]^ prepared a trifunctional Li_6.4_La_3_Al_0.2_Zr_2_O_12_ (LLAZO)‐modified interfacial layer, with the LLAZO coating protecting the electrolyte from direct contact with the cathode material. Meanwhile, the gradient doping of La^3+^ and Zr^4+^ into the LNMO lattice produced a Li‐La‐Al‐Zr‐O solid solution transition layer, which facilitated Li^+^ migration. The LLAZO coating could also restrict the growth of LNMO precursor particles and shorten the Li^+^ migration path. Notably, the modified material demonstrated excellent electrochemical performance, with a capacity retention of 72.6% after 600 cycles at 1C. Xiang Han et al.^[^ ^156^ ^]^ also introduced the Li_1.3_Al_0.3_Ti_1.7_(PO)_4_ (LATP) and Li_3_BO_3_ (LBO) bifunctional epitaxial layers (**Figure** **9**a,b) to create Li^+^ transport channels and enhance the material's mechanical stability to prevent particle rupture. Notably, the inert Li_3_BO_3_ coating inhibited TM dissolution and interfacial side reactions. Consequently, the material demonstrated excellent electrochemical cycling stability, with 89.7% capacity retention after 500 cycles at 200 mA g^−1^.

Using a polymer, Choi et al. adsorbed vanadium ionic complexes onto the surface of the material to form a Li_3_VO_4_ coating,^[^ ^157^ ^]^ as shown in Figure 9c. The Li_3_VO_4_ coatings promoted Li^+^ transfer and protected the active material from electrolyte erosion, yielding excellent high‐temperature cycling stability. In another study, Wonchang Choi et al. discovered that LiNbO₃ coatings effectively inhibited interfacial side reactions and improved Li⁺ diffusion kinetics.^[^ ^158^ ^]^ Furthermore, with minimal Nb entering the spinel structural lattice, LiNbO₃ coatings enhanced the material's structural stability and endowed it with excellent cycling performance. Additionally, Weikang Li et al. wrapped a lithium borate (LBO) epitaxial protective layer on the surface of LiNi_0_._5_Mn_1_._5_O_4_ using the mechanical dry‐mixing method.^[^ ^159^ ^]^ The wrapping induced BF_4_^⁻^ release, suppressing electrolyte salt decomposition and maintaining the uniform growth of positive and negative CEI/SEI. Consequently, the whole battery's cycling stability was effectively improved. Julia Maibach et al.^[^ ^160^ ^]^ further explored the effects of deionized water, as well as polyacrylic acid and phosphoric acid solutions, on the crystalline structure and elemental valence of the surface layer of the LiNi_0.5_Mn_1.5_O_4_ cathode. They found that Li^+^ dissolution occurred after treating LNMO with deionized water, resulting in a partial reduction of Mn^4+^ to Mn^3+^ on the surface. Following treatment with the polyacrylic acid solution, a protective layer of PAA was formed on the surface, decelerating Li^+^ dissolution. The second phase (MnPO_4_∙H_2_O) appeared after treatment with the aqueous phosphoric acid solution, resulting in a lower specific capacity.

Polymer coatings could increase the electronic conductivity of the spinel matrix, improve LNMO electrochemical properties, and reduce the bulk strain.^[^ ^161^ ^]^ Therefore, researchers improve its electronic conductivity to enhance its electrochemical properties. Yuliang Li et al. introduced a graphyne (GDY) protective layer at the interface of LiNi_0_._5_Mn_1_._5_O_4_,^[^ ^162^ ^]^ as shown in Figure 9d. According to the results, GDY effectively inhibited irreversible interfacial side reactions, scavenged HF, enhanced the structural stability of the material, and improved the long‐cycle stability and the battery's Coulombic efficiency. Manikandan Palanisamy et al. further created a polymethyl methacrylate (PMMA, wt.%) and polyvinylidene fluoride (PVdF, wt.%) polymer capping layer on the LNMO surface.^[^ ^163^ ^]^ This polymer facilitated the formation of Carbon Nanotube Conductive Networks (CNTs) on the surface of the material, enhancing its electronic and ionic conductivity and inhibiting the formation of the rock salt impurity phase (Li_x_Ni_1‐x_O_2_). Compared to the pristine samples, the modified samples demonstrated better electrochemical performance, with 82% capacity retention after 1000 cycles at 20C.

Low‐cost YPO_4_, which has stable chemical and physical properties and can form a dense and uniform coating on the surface of the electrode material, is the other coating commonly applied in LNMO. Besides its ability to withstand the electrode material's volume changes during charging and discharging, this coating is also not prone to cracking or peeling off, and could therefore enhance the material's structural stability.^[^ ^164^ ^]^ Xueliang Sun et al.^[^ ^165^ ^]^ reported that the AlPO_4_ coating could serve as a barrier between LNMO and the electrolyte, transform into a stable SEI layer during cycling, effectively prevent electrolyte decomposition and Mn dissolution, protect the LNMO structure during electrochemical reactions, and improve the electrochemical performance of LIBs. Furthermore, Yanrong Zhu et al. successfully applied FePO_4_ on the surface of LiNi_0_._5_Mn_1_._5_O_4_, coating it and avoiding the direct contact between the material and the electrolyte, ultimately preventing side reactions.^[^ ^166^ ^]^ The FePO_4_ coating reduced charge‐transfer impedance and improved the Li^+^ diffusion coefficient of the material. In other words, it improved LiNi_0_._5_Mn_1_._5_O_4_’s electrochemical activity, cycling stability, and rate performance. Additionally, Xueliang Sun et al. introduced the Li₃PO_4_‐TiO_2_ coating at the LNMO interface via ALD,^[^ ^167^ ^]^ as shown in Figure 9e. The coating functioned as a physical barrier and prevented interfacial side reactions while improving the material's ionic and electronic conductivity. Furthermore, it effectively suppressed structural degradation and TM dissolution on the cathode surface. Notably, the optimized LNMO demonstrated better rate performance and long‐cycle stability, with a capacity retention rate of 81.2% after 300 cycles.

Independently treating LNMO to transform its interface into two phases without adding other cladding layers could enhance interfacial stability while improving its electrochemical properties. Wang et al. hydrothermally modulated the LNMO interfacial phases to resolve Mn dissolution and improve the material's structural stability (Figure 9f).^[^ ^168^ ^]^ The solid‐phase reaction at the LNMO interface resulted in lamellar phases and Li/Mn antisite‐induced rock salt‐like phases. The Li/Mn antisite structure increased the Mn diffusion barrier, positively impacting Mn dissolution and Jahn‐Teller distortion. Furthermore, the inert rock‐like salt phase played a protective role. Notably, the rock salt‐like phase is unfavorable for charge transport, but the layered phase with good charge transport could make up for its deficiency, ensuring the material achieves structural stability and excellent charge transfer. Anmin Cao et al. introduced ZnO and reacted it with the material surface to form a biphasic layer comprising a rock salt layer and a lamellar phase.^[^ ^169^ ^]^ The rock salt‐like phase exhibited high Zn^2+^ levels, which could enhance the material's interfacial stability. Meanwhile, the layered structure created charge transfer channels, improving the material's electrochemical properties. Controlling the ratio of the two phases yielded a balance of improved material stability and electrochemical properties.

However, electrolyte additives can form a stable cathode electrolyte interface (CEI) on the positive electrode and a solid electrolyte interface (SEI) on the negative electrode, effectively mitigating lithium dendrite growth and electrolyte decomposition, enhancing the stability of electrode interfacial phases, and improving the battery's cycling performance. Kai Liu's team designed and synthesized a novel electrolyte additive, 2,2‐difluoroethylmethylsulfonic acid (FS).^[^ ^117^ ^]^ Unlike conventional additives, FS selectively adsorbs onto the manganese‐ and nickel‐containing transition metal sites of LNMO, forming a molecular “buffer layer” that inhibits high‐voltage side reactions of solvent molecules. In addition, the semi‐fluorinated ─CHF_2_ moiety in FS induces the formation of inorganic‐rich CEI and SEI layers, which effectively suppress LNMO particle cracking and transition metal dissolution. Weiwei Sun et al. proposed a ferrocene hexafluorophosphate (FHFP) electrolyte additive to enable dynamic Fe doping.^[^ ^16^ ^]^ Additionally, the additive molecules preferentially decompose at the cathode and anode interfaces, forming a thin and dense inorganic cathode electrolyte interface (CEI) and an F/P‐rich inorganic solid electrolyte interface (SEI), respectively. These interfaces effectively inhibit interfacial side reactions, suppress lithium dendrite growth, and stabilize the electrode interface. Jiulin Wang et al. designed a novel electrolyte with a wide electrochemical stability window, comprising a high‐fluorine‐content fluorinated phosphate ester (FEBFP) solvent and lithium (fluorosulfonyl) (nonafluorobutanesulfonyl) imide (Li[(FSO_2_)(n‐C_4_F₉SO_2_)N], LiFNFSI) salt.^[^ ^170^ ^]^ This electrolyte, featuring a wide electrochemical stability window (≈5.6 V), undergoes electrochemical reactions on the electrode surface to form inorganic‐rich, thermally stable, and dense SEI/CEI films containing LiF. These unique features enable LNMO to cycle for 200 cycles without capacity degradation. Ein‐Eli et al. developed an advanced high‐voltage electrolyte utilizing trifunctional additives.^[^ ^171^ ^]^ Theoretical calculations reveal that adding adiponitrile facilitates the presence of more hierarchical DFOB^−^ and PF_6_^−^ dual anion structures in the solvation sheath, leading to a faster de‐solvation of the Li cation. By combining fluorine‐ and nitrile‐based additives to produce a synergistic effect, they enabled the formation of stable inorganic SEI and CEI films, which inhibit lithium dendrite growth and suppress transition metal dissolution. Surface coating strategies for modifying LNMO are summarized in **Table** **3**.

**Table 3:** Summary of coating strategies to modify LNMO.

Coating material	Coating method	Cycling performance	Rate capability	Ref.
LaNiO_3_‐Li_3_PO_4_	Solid state	88.9 mAh g^−1^ after 200 cycles at 2C (capacity retention 91.8%)	94 mAh g^−1^ at 5 C	[172]
NiPO*_x_*	Wet chemical	97 mAh g^−1^ after 1000 cycles at 10C (capacity retention 95%)	102 mAh g^−1^ at 10C	[173]
FePO_4_	Sol–gel	117 mAh g^−1^ after 80 cycles at 2C	117 mAh g^−1^ at 2C	[166]
Al_2_O_3_	Pulsed laser deposition	111.2 mAh g^−1^ after 100 cycles at 1C (at 55 °C, capacity retention 84.1%)	120 mAh g^−1^ at 10C	[174]
Ta_2_O_5_	Solid state	122.3 mAh g^−1^ after 100 cycles at 55 °C (capacity retention 93%)	129.5 mAh g^−1^ at 0.2 C	[150e]
Fe_2_O_3_	Chemical Precipitation	122 mAh g^−1^ after 100 cycles at 1C (capacity retention 96%)	99 mAh g^−1^at 10C	[175]
YF_3_	Wet chemical	78 mAh g^−1^ after 100 cycles at 2C	95 mAh g^−1^ at 1C	[176]
CeF_4_	Hydrothermal	117 mAh g^−1^ after 100 cycles at 1C (capacity retention 97.6%)	120 mAh g^−1^ at 1C	[177]
Li_1.3_Al_0.3_Ti_1.7_(PO_4_)_3_	Solid state	132 mAh g^−1^ after 500 cycles at 1C (capacity retention 97.8%)	98.1 mAh g^−1^ at 10C	[178]
Li_1.4_Al_0.4_Ti_1.6_(PO_4_)_3_	Sol–gel	125.7 mAh g^−1^ after 500 cycles at 0.1C (capacity retention 68.9%)	102 mAh g^−1^ at 20C	[179]
Li_2_ZrO_3_	Wet chemical	125.8 mAh g^−1^ after 500 cycles at 5C (capacity retention 82.4%)	118.6 mAh g^−1^ at 10C	[180]
Carbon nanotubes	Solid state	95.5% capacity retention after 80 cycles at 1C	93 mAh g^−1^ at 8C	[181]

Overall, coating plays vital roles that include the following: maintaining interfacial stability between LNMO and the electrolyte; inhibiting the side reaction between LNMO and the electrolyte under high pressure; reducing the dissolution of Mn; and inhibiting the erosion of HF on the crystal structure of LNMO. Additionally, various coatings exhibit different modification effects. For instance, increasing the Li transport rate improves the rate performance, while increasing the electronic conductivity enhances the electrochemical performance. Similarly, slowing down the volumetric strain of the lithium ion de de‐insertion/insertion process improves the structural stability of the material.

### 3.3 Morphology and Size Management

Electrode materials primarily facilitate Li⁺ and electron exchange through the surface or near‐surface portion of the material during charge/discharge. Subsequently, nanoscale particle‐sized electrodes or electrodes exhibiting a high‐specific‐surface‐area structure present an effective strategy to improve electrochemical performance.^[^ ^182^ ^]^ The small size of nanostructured electrode materials, in conjunction with the shorter Li⁺ transport paths, improves the Li‐ion transport rate. Additionally, the nano‐size provides a buffer mechanism to the stress generated by the volume expansion/contraction of the Li⁺ de/embedding process, thereby preventing the material from rupturing or generating microcracks.^[^ ^183^ ^]^ Materials with 1D nanostructures (nanowires, nanorods, nanotubes, etc.) possess the capability to shorten the Li⁺ de‐insertion/insertion migration paths and provide directional transport channels for electrons, in addition to activating more active reaction sites.^[^ ^184^ ^]^ The 2D structure of the material exhibits a larger specific surface area, subsequently enhancing the reaction area and shortening the Li⁺ transport distance to improve both the material's rate of performance and cycling.^[^ ^48^ ^]^ The outer shell of the core–shell structure protects the inner core, thereby endowing the core material with enhanced structural stability and thermal stability. Additionally, the gap between the outer shell and the inner core serves as a buffer for lattice strain induced by the Li⁺ de‐/insertion process. This not only prevents the material's structure from collapsing during long‐term cycling but also shortens the transport path of Li⁺.^[^ ^185^ ^]^

**Figure 10:**
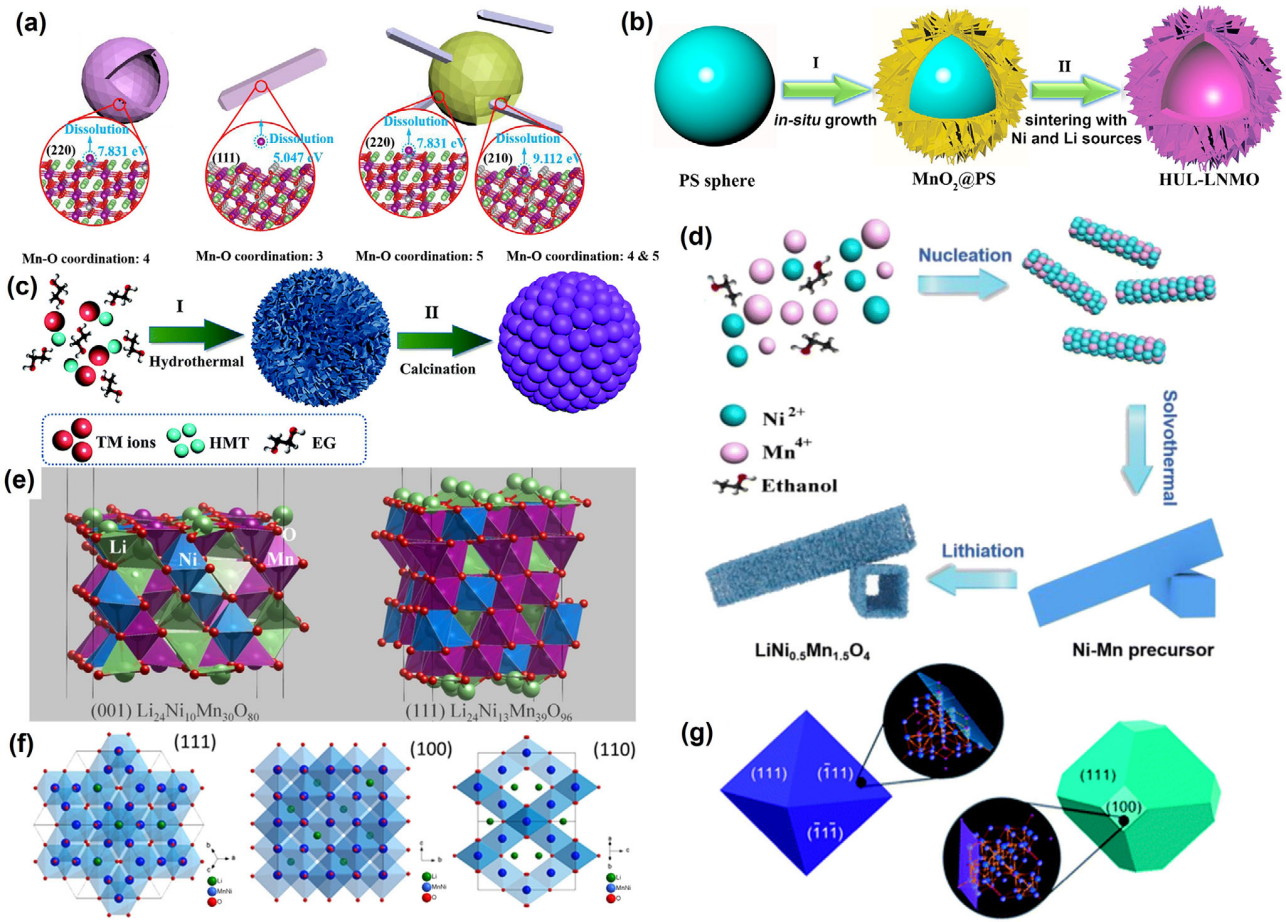
a) Isotropic Surfaces of Spherical, Nanorod, and Hybrid‐Structured LNMO. Reproduced with permission.^[^ ^186^ ^]^ Copyright 2019, Elsevier Inc. b) Schematic synthesis of layered sea urchin‐like LNMO hollow spheres. Reproduced with permission.^[^ ^187^ ^]^ Copyright 2018, Elsevier Inc. c) Schematic diagram showing the synthesis process of layered poplar‐like LNMO. Reproduced with permission.^[^ ^188^ ^]^ Copyright 2018, The Royal Society of Chemistry. d) Schematic of the synthesis of layered hollow microcubic LNMO. Reproduced with permission.^[^ ^189^ ^]^ Copyright 2019, Springer. e) LNMO (001) and (111) Surface symmetric nonmetric models. Reproduced with permission.^[^ ^191^ ^]^ Copyright 2018, The Electrochemical Society. f) Schematic illustration of atoms with different surface orientations. Reproduced with permission.^[^ ^192^ ^]^ Copyright 2016, American Chemical Society. g) Schematic diagram of Li⁺ arrangement on the crystal faces of octahedron (111) and truncated octahedron (100). Reproduced with permission.^[^ ^193^ ^]^ Copyright 2013, The Royal Society of Chemistry.

Nanostructured LNMO materials, including nanoparticles (0‐D), nanofibers (1‐D), nanorods (1‐D), and nanoplates (2‐D), have been extensively explored, with reports indicating that they can be generated to exhibit excellent electrochemical properties. Guo et al. designed LNMO materials with a spherical‐nanorod structure, characterized by specific crystallographic facets,^[^ ^186^ ^]^ as shown in **Figure** **10**a. The structure facilitates greater Li─O bonding to facilitate Li^+^ transport. The relatively short Mn─O bond length helps to stabilize the spinel structure, thereby suppressing the bulk strain of the material during charging and discharging. Additionally, the exposed (210) crystal surface with a high density of Mn defects can inhibit manganese dissolution, improving the cycling stability of the material. Consequently, following 1000 cycles at 10C, a capacity of 107.8 mAh g⁻¹ persists. Chunman Zheng et al. synthesized a hierarchical porous sea urchin‐like hollow sphere structure of LiNi_0_._5_Mn_1_._5_O_4_,^[^ ^187^ ^]^ as shown in Figure 10b. This structure exhibits excellent ability to shorten the Li^+^ diffusion path and reduce the volumetric strain during the charge/discharge process. Additionally, due to its exposed (111) crystal surface and unique hollow structure, it effectively inhibits manganese dissolution, thereby enhancing both the materials' cycling stability and performance rate. After 1500 cycles at a high rate of 30C, it exhibits capacity retention as high as 92%. Kai Xie et al. prepared a bayberry‐like LiNi_0_._5_Mn_1_._5_O_4_ structure,^[^ ^188^ ^]^ as shown in Figure 10c. This structure was characterized by a porosity that effectively alleviates the volumetric strain during the process of high‐rate charging and discharging. The inherent hierarchical microporous/nanostructures can promote the diffusion of lithium ions, conferring the material with superior electrochemical performance. Despite being subjected to 1200 cycles at 30C, the material exhibited a capacity retention rate of 84%. Luo et al. prepared hollow micro‐rectangular LiNi_0_._5_Mn_1_._5_O_4_ using a simple solvothermal and lithiation process (Figure 10d).^[^ ^189^ ^]^ This structure exhibited excellent ability to diffuse Li⁺ due to its hierarchical hollow structure, in addition to offering pore space to alleviate the volume expansion during charging and discharging. Guangchuan Liang et al. synthesized LNMO cathode materials with a hollow hierarchical structure using the urea‐assisted hydrothermal method.^[^ ^190^ ^]^ The hollow hierarchical structure provides additional active sites and a shorter Li⁺ diffusion path. Additionally, the hollow structure buffers the volume change and alleviates the mechanical strain caused by repeated Li⁺ removal/embedding. The prepared LNMO materials exhibit superior electrochemical properties, with 96.8% capacity retention after 100 cycles at 1C and 124.9 mA h g⁻¹ discharge capacity at 10C. Minggao Ouyang et al. explored the inherent stability of single‐crystal secondary particles LiNi_0.5_Mn_1.5_O_4._^[^ ^51^ ^]^ In situ XRD and HRTEM showed that polycrystalline LiNi_0.5_Mn_1.5_O_4_ exhibited a significantly pronounced two‐phase transition from Li_1‐x_Ni_0.5_Mn_1.5_O_4_ to MnO_2_ during high‐voltage delithiation/lithiation, and the accumulation of lattice strains induced the generation of microcracks. These events resulted in capacity degradation, whereas the single‐crystal particles exhibited homogeneous solid solution reaction and excellent electrochemical cycling stability.

Overall, the varying morphological structures of LNMO exhibit different effects on its electrochemical performance. Single‐crystal LNMO particles possess longer Li⁺ de‐insertion/insertion diffusion paths and larger migration barriers. Conversely, nano‐sized LNMO can reduce the Li⁺ migration barrier in the lattice, with it high specific surface area providing more sites for electrochemical reactions, thereby improving its rate of performance. However, the high specific surface area affects its cycling stability since it exacerbates side reactions with the electrolyte. Concurrently, its lower tap density results in a lower volumetric energy density. LNMO particles with nano‐hierarchical structures exhibit features similar to those of nanostructures. Their porous or hollow structures provide interconnected ion diffusion channels that can relieve strain, thus reducing the collapse of the crystal structure.

### 3.4 Surface Orientation Management

The orientation of LNMO crystal surfaces has a significant influence on the material's structural stability, lithium‐ion diffusion kinetics, and manganese solubility of the material. The (111) crystal faces of LNMO have the densest ionic arrangement, the lowest surface energy, and the lowest manganese solubility.^[^ ^194^ ^]^ Therefore, exposing more (111) crystal faces during LNMO preparation helps to mitigate the harmful side reactions between the electrode surface and the electrolyte, increase the Li and e⁻ transport rates, and improve the electrochemical performance of the material.^[^ ^33^, ^195^ ^]^ Vitaly Alexandrov et al. investigated the stability of Mn and Ni on the (001) and (111) crystal surfaces of LNMO cathode materials using Density Functional Theory (DFT) calculations and ab initio molecular dynamics (AIMD)simulations (Figure 10e).^[^ ^191^ ^]^ They revealed that the protonation of surface oxygen atoms and oxygen vacancies contributes to the reduction of surface transition metals. In the fully discharged (lithiated) state, the charge transfer between Ni and Mn on the (001) crystal surface reduces the surface Mn oxidation state, exhibiting Mn(III)/Mn(IV) and Ni(II)/Ni(III) oxidation states, which facilitates the dissolution of transition metals. In contrast, Ni(III) and Mn(IV) can only be observed on the (111) crystal surface, inhibiting the dissolution of transition metals. Jie Li et al. studied the influence of different morphologies (crystal plane orientations and particle sizes) on kinetic parameters (electronic conductivity and Li⁺ diffusion rate) (Figure 10f).^[^ ^192^ ^]^ Their finding revealed that an appropriate crystal plane orientation can simultaneously provide the compatibility of structural stability and long‐term cycling stability. In contrast, the truncated octahedral materials with (100) crystal planes and micrometer‐sized particles (3–5 µm) exhibit good long‐term cycling stability. This material exhibited a capacity retention rate of 77% after 2000 cycles at 1C. The rate performance and cycling stability of the common octahedrons composed of (111) crystal planes only exhibit a suboptimal feature. In contrast, materials with a medium particle size (≈1 µm) and a small number of (100) crystal planes have a higher Li⁺ diffusion coefficient. Therefore, they possess a superior rate of performance and cycling stability. Arumugam Manthiram et al.^[^ ^193^ ^]^ investigated the influence mechanism of the nucleation and growth of crystals in different processes during the co‐precipitation preparation on the secondary particle morphology (Figure 10g), the degree of ordering between Mn⁴⁺/Ni^2^⁺, the Mn^3^⁺ content, the surface formation energy, as well as the variation of lattice parameters with the charge state. They demonstrated that in the spinel structure, the (100) planes exhibit a sparsely dense ion arrangement and higher surface energy, which are prone to induce the dissolution of transition metal elements. Conversely, the (111) planes have the densest ion arrangement and the lowest surface energy, necessary to which is necessary to inhibit Mn dissolution.

Although the crystallographic orientation has exhibits significant impact large influence a large influence on the electrochemical properties of LiNi_0.5_Mn_1.5_O_4_ materials, the roles of the (111) and (100) crystal planes remain incompletely understood and controversial. This is attributed to the numerous factors influencing the electrochemical properties of LiNi_0.5_Mn_1.5_O_4_ materials, such as the crystal structure, the content and distribution of the impurity phase LiNi_1‐y_O, and the particle size, as well as the orientation of the crystal plane. Therefore, future studies should focus on eliminating confounding and other existing variables besides crystal plane orientation to analyze the mechanism by which crystal plane orientation affects the electrochemical properties of LiNi_0.5_Mn_1.5_O_4_ materials.

## 4 Summary

Cobalt‐free LNMO cathode materials exhibit promising potential, but their large‐scale application faces challenges. This review elaborates on the decay mechanisms of LNMO, including irreversible phase transition, cation migration, transition metal dissolution, intergranular cracking, interfacial reaction, and loss of reactive oxygen species. Additionally, it summarizes the corresponding modification strategies, including bulk phase doping, interfacial engineering, morphology modulation and crystal surface engineering. Specifically, crystal surface modulation is based on the ion diffusion equation to reduce the transport paths of lithium ions and electrons, thus improving the electrochemical performance of electrode materials. However, the easy aggregation of nanostructures, the low tap density, and the uncontrollable side reactions between the material and the electrolyte warrant in‐depth exploration. Surface coatings or composite structures can improve the conductivity of the electrode material, enhance the interfacial stability between the electrode and the electrolyte, and inhibit the dissolution of transition metals and erosion by HF. However, achieving uniform coating at the interface of electrode materials is extremely challenging. Elemental doping can facilitate lithium‐ion transport and improve the electrochemical properties of materials by altering crystal – bond strength and local environment, enhancing backbone stability, and changing atomic spacing. Nonetheless, doping typically reduces the amount of active material and lowers the capacity, especially the initial capacity. Additionally, it is crucial to explore the compatibility of the ionic radii and valence states of the dopant elements with those of the bulk‐phase ions. Synergistic modification through the surface coating, ion doping, morphology modulation, and crystal surface modulation techniques may contribute to enhancing the electrochemical performance of LNMO in the future. Based on the above findings, future research on the development of LNMO may focus on material design, in situ characterization techniques, and machine and theoretical determination.

### 4.1 Material Design

To meet future commercial needs, it is crucial to explore the design principles and synthesis methods of stabilized LNMO to lay a solid foundation for practical applications.

#### 4.1.1 Structure Optimization

Explore different site doping, such as directional substitution of manganese sites, to achieve precise regulation of manganese valence state, inhibition of transition metal dissolution, and Jahn‐Teller effect. Introducing vacancies can help inhibit the migration and dissolution of manganese ions and enhance the structural stability of the material. Although surface modification can improve the interfacial stability of materials, there is still a lack of sufficient studies on this approach by researchers. In‐depth studies on surface modification strategies and the selection of suitable cladding materials to improve material stability are essential. In addition, conduct an in‐depth exploration of the relationship between disorder and performance, develop new types of disordered cathode materials, devise synthesis methods to control disorder, improve the electrochemical performance of the materials, precisely control the valence state of manganese, promote the stable structure of the materials, and inhibit the Jahn‐Teller effect as well as the migration and dissolution of manganese ions.

#### 4.1.2 Electrolyte Optimization

LNMO exhibits a high operating voltage plateau, while conventional electrolytes exhibit suboptimal stability at high voltages and are prone to decomposition and generate HF to corrode the cathode material. In addition, at high voltage, interfacial side reactions lead to the formation of unstable electrolyte interfaces (CEI/SEI films), exacerbating electrode capacity decay. Notably, it is important to design and develop an interface composition of high‐voltage and safe electrolyte conditioning to form a thin and dense inorganic cathode electrolyte interface (CEI) and an inorganic solid electrolyte interface (SEI) rich in F and P. Positively, the CEI formed can effectively inhibit the side reaction between the electrolyte and the positive material, reduce the dissolution of transition metal ions, and inhibit the decomposition of the electrolyte at the same time. Negatively, the SEI formed can effectively inhibit the growth of lithium dendrites and improve the stability of the negative electrode and the safety of the battery. The stabilized interface formed at the same time reduces the interfacial impedance. Therefore, the development of new electrolytes with high voltage is crucial to improve LNMO materials.

#### 4.1.3 Establishing Structure–Performance Relationships

Establishing structure–performance relationships is crucial for guiding the production of LNMO and thus expanding the horizons of LIBs design. Currently, the study of the structure–performance relationship is not deep enough, and the relevant performance of different space group structures varies greatly. Therefore, it is necessary to further investigate the effect of material composition on structure and ultimately elucidate the structure–performance relationship.

### 4.2 There is a Need to Elucidate the Reaction Mechanisms Using Advanced Characterization Techniques

The electrochemical performance of cathode materials is closely related to the internal structural and compositional changes associated with the process of detaching/embedding lithium ions. A comprehensive understanding of their physical/chemical changes throughout the charging/discharging process is necessary to realize high‐performance LIBs. However, conventional techniques are deficient in precisely monitoring of accurately monitoring information, especially phase transitions and interfacial structure and composition at high voltages and temperatures. The formation mechanisms of CEI/SEI films and the vacancy induction mechanism are probed using reliable in situ and variable temperature characterization techniques. These techniques include in‐situ synchrotron radiation X‐ray diffraction, in situ transmission electron microscopy, and in situ infrared spectroscopy. Furthermore, researchers investigate the constitutive relationship between novel disordered cathode materials and their electrochemical performance using corresponding geometric phase analysis (GPA).

### 4.3 Machine Learning and Theoretical Computing

The combination of theoretical computation and machine learning can enhance the in‐depth investigation of LNMO attenuation and improvement mechanisms. For example, integration of artificial intelligence and machine learning has facilitated the prediction and optimization of material synthesis conditions, cladding materials, novel electrolytes, doping elements, and sites to refine the material design. Additionally, molecular dynamics simulations and first‐principles calculations hold significant potential in providing detailed insights into structural stability and ion de/embedding at the molecular and atomic levels, respectively. Moreover, researchers can utilize DFT calculations to elucidate the interfacial structure of the materials and the mechanism of component evolution.

## Conflict of Interest

The authors declare no conflict of interest.
